# Dipeptidyl peptidase‐4 deficiency prevents chronic stress‐induced cardiac remodeling and dysfunction in mice

**DOI:** 10.1096/fj.202402328R

**Published:** 2025-02-19

**Authors:** Zhe Jiang, Longguo Zhao, Minglong Xin, Ying Wan, Shengnan Xu, Xueling Yue, Xianglan Jin, Rihua Cui, Yanglong Li, Weon Kim, Hongxian Wu, Xian Wu Cheng

**Affiliations:** ^1^ Department of Cardiology and Hypertension, Jilin Provincial Key Laboratory of Stress and Cardiovascular Disease Yanbian University Hospital Yanji Jilin China; ^2^ Department of Cardiology The Second Hospital of Jilin University Changchun Jilin China; ^3^ Department of Anesthesiology Yanbian University Hospital Yanji Jilin China; ^4^ Department of Radiology Yanbian University Hospital Yanji Jilin China; ^5^ Division of Cardiology, Department of Internal Medicine Kyung Hee University Hospital, Kyung Hee University Seoul Republic of Korea; ^6^ Department of Cardiology, Zhongshan Hospital Fudan University, Shanghai Institute of Cardiovascular Diseases, National Clinical Research Center for Interventional Medicine Shanghai China; ^7^ Department of Community Healthcare and Geriatrics Nagoya University Graduate School of Medicine Nagoya Japan

**Keywords:** apoptosis, cardiac injury, chronic stress, fibrosis, inflammation, oxidative stress

## Abstract

Exposure to chronic psychosocial stress is a risk factor for metabolic cardiovascular disorders. Dipeptidyl peptidase‐4 (DPP‐4) plays essential roles in human pathobiology, and we recently showed that DPP‐4 levels are increased by chronic stress in murine models. We here investigated the role of DPP‐4 in stress‐related cardiac injury and dysfunction in mice, focusing on oxidative stress and cardiac apoptosis. Male mice were randomly assigned to non‐stress and two‐week immobilized‐stress groups for biological and morphological studies. On day 14 post‐stress, stress had increased blood pressure, heart weight, cardiac myocyte size, and interstitial fibrosis, impaired cardiac diastolic function, and increased plasma levels of DPP‐4 and glucose. The stressed mice also had increased levels of monocyte chemoattractant protein‐1, inteleukin‐6, gp91^phox^, matrix metalloproteinase‐2 (MMP‐2), MMP‐9, tissue inhibitor of MMP‐1/−2, caspase‐8, and Bax genes and/or proteins and lowered levels of Bcl‐2, p‐Akt, and endothelial nitric oxide synthase (eNOS) proteins. DPP‐4 inhibition by either a genetic or pharmacological approach ameliorated the stress‐induced targeted molecular and morphological changes. In vitro, DPP‐4 inhibition also mitigated the alterations in the targeted caspase‐8, Bcl‐2, eNOS, and p‐Akt proteins in H9c2 cardiomyocytes in response to H_2_O_2_. DPP‐4 inhibition appeared to improve the stress‐induced cardiac injury and dysfunction in mice, possibly via the improvement of oxidative stress and apoptosis, suggesting that DPP‐4 could become a novel therapeutic target for chronic psychological stress‐related metabolic cardiovascular disorders.

## Graphical abstract

Genetic and pharmacological interventions against dipeptidyl peptidase‐4 ameliorate cardiac injury and dysfunction in mice under chronic stress conditions.

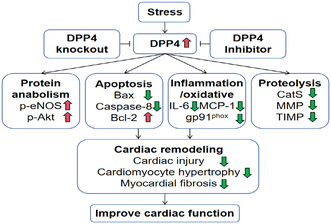

## 1 INTRODUCTION

Cardiovascular disease (CVD) is a leading global cause of mortality, with stress recognized as a contributing factor to their development.^1^, ^2^ Extensive research has been conducted to explore the relationship between chronic stress and various health risks, including various cancers^3^, ^4^ and CVD^5^; in regard to CVD, chronic stress has been shown to increase the CVD‐associated mortality rate.^5^, ^6^, ^7^

Stress activates the neuroendocrine system, including the hypothalamic–pituitary–adrenal (HPA) axis and the sympathetic nervous system.^8^ Prolonged stress will lead to stronger stimulation of the sympathetic nervous system, resulting in sustained elevated levels of stress hormones, which in turn trigger impairment of cardiovascular, immune, metabolic, and neurological function.^9^ Chronic stress can disrupt the balance of the immune system, intensify inflammation, diminish immune surveillance, and consequently contribute to the development of various related diseases.^10^ Additionally, chronic stress has been associated with endothelial dysfunction, changes in vascular reactivity, and enhanced coagulation, although the mechanisms remain incompletely understood.^11^, ^12^, ^13^

Dipeptidyl peptidase‐4 (DPP‐4) is a serine protease widely distributed in cell membranes and is capable of degrading a variety of substrates, including glucagon‐like peptide‐1 (GLP‐1) and glucose‐dependent insulinotropic peptide (GIP).^12^ Recent studies have shown that DPP‐4 is widely expressed in the cardiovascular system, including in endothelial cells and cardiomyocytes,^10^, ^14^, ^15^, ^16^ suggesting that it may be involved in the onset and progression of CVD. Currently, DPP‐4 inhibitors are widely used as a type of hypoglycemic agent in diabetes treatment, mainly by increasing the concentration of GLP‐1 and GIP in pancreatic β‐cells and promoting insulin secretion, thus effectively controlling blood glucose.^17^ Interestingly, it has been found that DPP‐4 inhibitors also exhibit protective effects against metabolic CVD,^18^, ^19^ but their role in heart failure remains controversial.^20^, ^21^, ^22^ It was reported that chronic stress increases DPP‐4 activity in plasma and tissues of mice and rats.^23^, ^24^, ^25^ Accumulating evidence indicates that pharmacological and genetic and pharmacological interventions targeted toward DPP4 ameliorate vascular aging, angiogenesis, and atherosclerosis under chronic stress conditions in these animal models.^26^, ^27^, ^28^ However, the current understanding of the mechanisms of cardiac protection induced by DPP‐4 under stress remains incomplete.

Accordingly, we conducted this study to investigate the possible protective role of DPP‐4 in stress‐induced CVD. We investigated the role and mechanism of DPP‐4 in stress‐induced CVD by using DPP‐4 knockout mice, the DPP‐4 inhibitor anagliptin, and a stress model. To further validate our findings, relevant cellular experiments were performed. For this purpose, we utilized an H_2_O_2_‐induced cell damage model and investigated the signaling pathways through the addition or omission of anagliptin as well as PI3K/AG490.

## 2 MATERIALS AND METHODS

### 2.1 Animals

Eight‐week‐old male DPP4^+/+^ (C57BL/6J) mice and DPP4^−/−^ (DPP4 knockout) mice^23^ weighing 22–26 g were used. Mice were placed individually in cages to ensure they had ready access to food and water. The environment was maintained with a 12‐h light/dark cycle (light on at 7:00 A.M.), a temperature of 22–24°C, and 50% ± 10% humidity. All experimental protocols (Figure S1A) were approved by the Institutional Animal Care and the Use Committee of Yanbian University (protocol no. YD20211120005).

### 2.2 Mouse model of chronic‐restraint stress and mouse grouping

Mouse immobilization stress model (Figure S1B) was established via the application of chronic‐restraint stress.^29^ First, to assess the effect of chronic stress on myocardial injury, DPP4^+/+^ mice were subjected to two weeks of restraint stress as previously described.^23^ To prevent the mice from becoming accustomed to the restraint stress, the mice were subjected to several different combinations of stressors over each week (Mondays to Sundays) and altered the stress randomly as follows: (1) overnight illumination: The mice were placed separately in an immobilization stress tube in a room with all‐night lighting (from 20:00 to 8:00); (2) cage damp and horizontal: We removed the sawdust and replaced some water in the cage, and then suspended the immobilization stress tube horizontally so that the mouse's tail was in the water (4 h/time); (3) Cage tilt: We put the mouse into immobilization stress tube and suspended the cage at a 45° angle (4 h/time). During the 4‐h restraint period, animals were restricted from food and water intake. The control group mice were allowed to move freely in their individual cages. Each animal's weight, blood pressure (BP), and blood glucose were checked every week. In a separate DPP‐4 inhibition experiment, mice were randomly divided into two groups and received daily oral gavage of either distilled water (100 μL) or the DPP4 inhibitor (anagliptin, 30 mg/kg/100 μL per day), while undergoing chronic‐restraint stress for 4 h each day over a two‐week period. In a separate DPP‐4 genetic inhibition experiment, both DPP4^+/+^ and DPP4^−/−^ mice were randomly subjected to the same 4‐h chronic‐restraint stress for a duration of two weeks, whereas both genetic mice were allowed contact with each other and left undisturbed as the corresponding controls.

In this study, male DPP4^+/+^ and DPP4^−/−^ mice were utilized. Each group consisted of six mice, randomly divided into six groups: the control groups (NS‐DPP4^+/+^ and NS‐DPP4^−/−^), the stress groups (S‐DPP4^+/+^ and S‐DPP4^−/−^), the stress treated with vehicle (S‐DPP4^+/+^‐Veh), and anagliptin groups (S‐ DPP4^+/+^‐Ana).

### 2.3 Sample collections

For tissue collection, mice were anesthetized with isoflurane and their blood was collected by cardiac puncture. Serum and the heart and adipose were collected. A portion of each type of tissue, including the heart tissue, was quickly frozen at −80°C for further use. The remaining portion of the heart was used for histopathological examination or kept in RNAlater solution for the targeted gene assays.

### 2.4 Plasma DPP4 level analysis

Plasma DPP4 activity was assayed using the DPPIV‐Glo Protease Assay (Promega, Madison, WI), according to the manufacturer's instructions. Human recombinant DPP4 from Sigma‐Aldrich (St. Louis, MO) was utilized to construct a standard curve. Luminescence intensity was measured using a POWERSCAN4 instrument (BioTek Instruments, Winooski, VT), and the anagliptin‐sensitive value (i.e., the absolute difference between the absence value and the presence value of DPP4 activity) in relative light units per mL of plasma was calculated with the standard curve to represent the DPP4 level (ng/mL).

### 2.5 Real‐time polymerase chain reaction (RT‐PCR)

Total RNA was extracted by using an RNeasy Mini Kit (Qiagen, Hilden, Germany) following the manufacturer's protocol.^30^ The quantitative real‐time polymerase reaction chain (RT‐PCR) analysis was performed with primers specific for gp91^phox^, monocyte chemoattractant protein‐1 (MCP‐1), metalloproteinase‐2 (MMP‐2), MMP‐9, tissue inhibitor of metalloproteinase‐1 (TIMP‐1), TIMP‐2, cathepsin S (CatS), and interleukin‐6 (IL‐6) with the use of an ABI 7300 PCR System (Applied Biosystems, Foster City, CA). The expression of glyceraldehyde 3‐phosphate dehydrogenase (GAPDH) was measured in parallel to that of the genes of interest and was used as an internal standard for the quantitative comparison of mRNA levels. The primer sequences are listed in Table S1.

### 2.6 Western blot analysis

The proteins used for Western blotting were extracted using RIPA lysis buffer (Solarbio, Beijing, China) supplemented with protease inhibitors (Solarbio). The proteins were quantified using a BCA Protein Assay Kit (Solarbio). The same amounts of protein (40 μg) were loaded and separated by sodium dodecyl sulfate‐polyacrylamide gel electrophoresis (SDS‐PAGE). The membranes were incubated overnight with primary antibodies against endothelial nitric oxide synthase (eNOS) (1:1000; BD Biosciences, San Jose, CA), phospho‐eNOS (p‐eNOS^S1177^, 1:1000; Abmart, Shanghai, China), p‐Akt^473^ (1:1000; Cell Signaling Technology, Beverly, MA), gp91^phox^ (1:1000; BD Biosciences), caspase‐8 (1:1000; Cell Signaling Technology), Bax (1:1000; Cell Signaling Technology), and Bcl‐2 (1:1000; Cell Signaling Technology) and then incubated with the related secondary antibodies at a 1:2000–5000 dilution. Targeted proteins were analyzed using an Amersham ECL Prime Western Blotting Detection kit and visualized using an Azure 500 Bioanalytical Imaging System. The levels of the targeted proteins quantified by Western blots were normalized by loading GAPDH levels.

### 2.7 Histopathological examination

Fresh heart samples were collected. The samples were fixed in 4% paraformaldehyde, embedded in paraffin, and stained with hematoxylin and eosin (H&E) or Masson's trichrome kit (Solarbio).^31^ The sections were examined under a light microscope and photographed. In addition, a color image analysis system (ImageJ) was used to calculate the ratio of the fibrotic area to the whole area.

### 2.8 Blood pressure measurements

BP was measured using a noninvasive tail‐cuff BP system (BP‐2000; Visitech Systems). Briefly, mice were placed into a plastic tube restrainer, BP recording cuffs were placed over the tail, and the mice were allowed to adapt to the restrainer for 5 min. BP was then measured for 10 acclimation cycles followed by 25–30 measurement cycles. Heating pads were used to keep the mice warm throughout the experiment to ensure sufficient blood flow to the tail.^32^

### 2.9 Measurement of blood glucose levels

Blood glucose levels were measured at 2 h after stress by tail vein blood collection in mice. For all experiments, the blood glucose of mice was determined with a blood glucose meter (YUWELL, China) from the tail vein blood.

### 2.10 Intraperitoneal glucose and insulin tolerance tests

At day 14 after daily restrain stress, the mice were applied to insulin tolerance and intraperitoneal glucose tests (ITTS and GTTs) according to the manufacturer's instructions. In brief, for ITT, following being fasted for 16 h, mice were intraperitoneally injected with insulin (0.75 units/kg; Actrapid Penfill, NovoNordisk), and blood glucose was evaluated. For GTT, after fasted for overnight, the mice‐loaded D‐glucose (2 g/kg, Sigma‐Aldrich, St. Louis, MO) was subjected to monitor blood glucose up to 120 min with a blood glucose level monitor (Glutest Ace; Sanwa Kagaku Kenkyusho Co., Nagoya, Japan).

### 2.11 Echocardiography

After two weeks of stress, animals in each group were anesthetized with 1%–2% isoflurane (inhalation), and cardiac function was measured by transthoracic echocardiography (Vevo 2100; Visualsonics, Shanghai, China). An M‐mode image of the left ventricle in the parasternal long‐axis view was captured. Data from at least three cardiac cycles were collected. Diastolic function was measured by PW Doppler imaging including the isovolumetric relaxation time (IVRT), isovolumetric contraction time (IVCT), ratio of peak velocity of early to late filling of mitral inflow (E/A), and ejection time (ET). The myocardial performance index (MPI) was calculated as (IVCT + IVRT)/ET.^33^

### 2.12 Plasma corticosterone level analysis

The levels of mouse plasma corticosterone were examined at a commercial laboratory (SRL, Tokyo, Japan).^34^

### 2.13 TUNEL assay

Apoptosis in H9c2 cells was assessed by terminal transferase UTP nick end labeling (TUNEL) assay.^32^ Briefly, cells were treated with different concentrations of H_2_O_2_ (0, 200, 400, and 600 μmol/L) for 24 h and then fixed with 4% paraformaldehyde for 30 min at room temperature. TUNEL staining was performed using a Cell Death Detection kit (Beyotime Biotechnology, Shanghai, China). The index of apoptosis was calculated using the formula ([number of TUNEL‐positive cells/total number of cells] × 100%).

### 2.14 Cell culture and treatment

The H9c2 (2‐1) (Procell CL‐0089) cell line was kindly provided by Procell Life Science & Technology (Wuhan, China). H9c2 cells from passages 4 to 6 were used for all experiments. H9c2 cardiomyocytes were maintained in Dulbecco's modified Eagle's medium (DMEM) supplemented with 10% fetal bovine serum (FBS) and 1% penicillin‐streptomycin solution at 37°C in 5% CO_2_. During the experiment, the medium was replaced with the medium without serum for 12 h.

1. Initially, cells were seeded in serum‐free media and exposed to varying concentrations of H_2_O_2_ (0, 200, 400, and 600 μM) for 24 h, followed by Western blot analysis to assess the expression of eNOS.
2. Cells were divided into four groups and then pre‐treated with 0, 10, 20, or 30 μM of anagliptin for 30 min. The pre‐treated cells were treated with H_2_O_2_ (400 μM) for 24 h to assess the protein expression of eNOS, caspase‐8, and Bcl‐2.
3. To elucidate the mechanism underlying the effects of DPP4 inhibitors on eNOS/p‐Akt expression under oxidative stress, H9c2 cells were pre‐treated with or without two inhibitors: the PI3K/AKT inhibitor (LY294002; 20 μM) and the JAK/STAT3 pathway inhibitor (AG490; 5 μM) (both from APExBIO Technology, Houston, TX). After 24 h of serum‐free incubation in the presence or absence of anagliptin (30 μM) and/or H_2_O_2_ (400 μM), eNOS and p‐Akt^473^ proteins were detected by Western blotting. These cellular experiments were conducted at least three times to ensure the robustness and reliability of the results.

### 2.15 Statistical analysis

All data are expressed as mean ± standard error of the mean (SEM). We performed a one‐way analysis of variance (ANOVA) for comparisons of multiple groups followed by Tukey's post‐hoc test or Student's *t*‐test for comparisons of two independent sample groups with GraphPad Prism version 9.0.0 software. The cardiac morphological analyses were evaluated by two observers in a blind manner, and the values they obtained were averaged. Probability (*p*)‐values <.05 were considered significant.

## 3 RESULTS

### 3.1 Effects of chronic stress on body weight, adipose tissue weight, the ratio of heart weight to tibia length, blood pressure, and blood glucose

**FIGURE 1:**
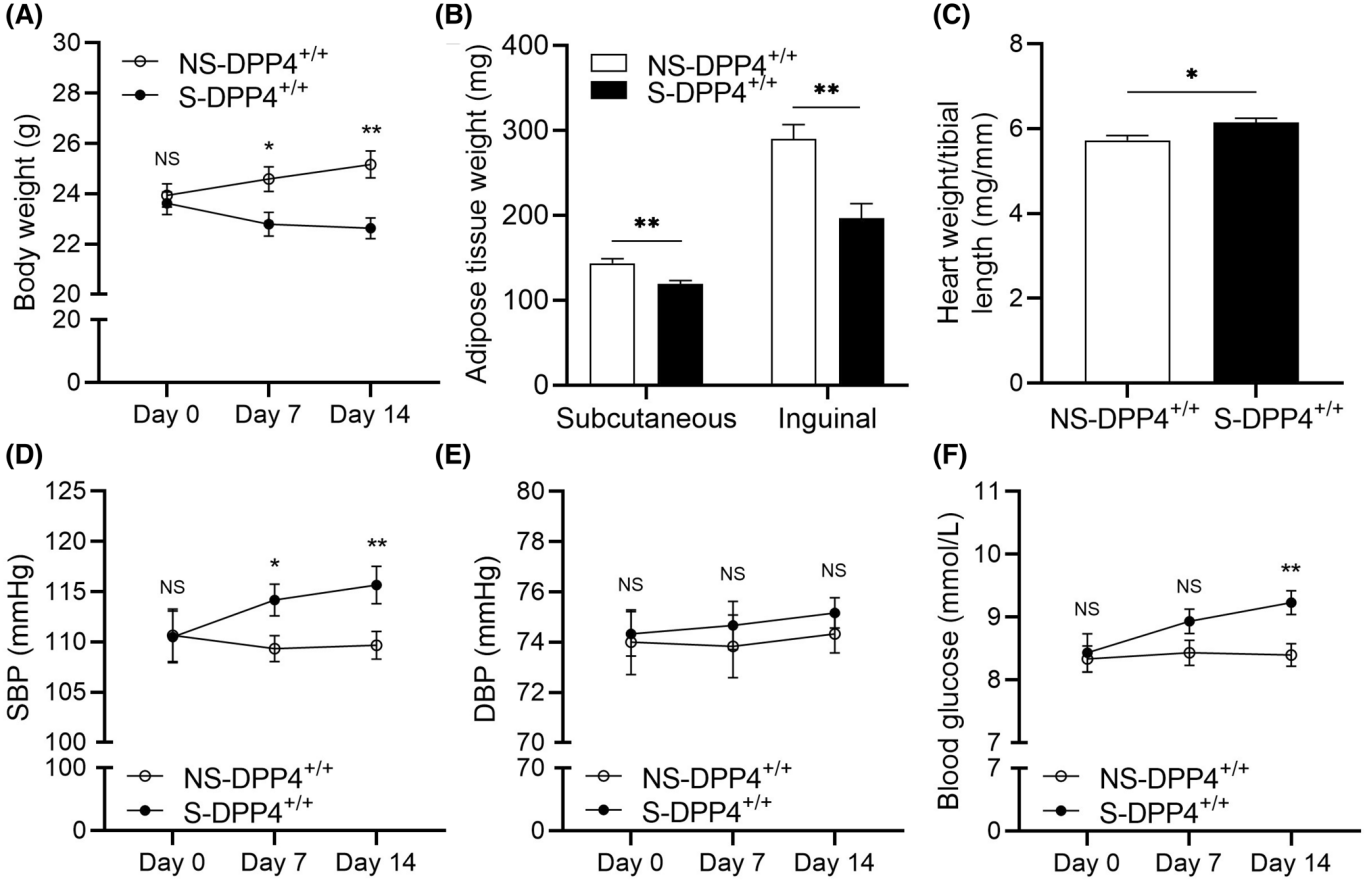
Changes in various parameters under conditions of chronic stress exposure. (A) Body Weight (BW). (B) Subcutaneous and inguinal adipose tissue weight. (C) Quantification of the heart weight to tibia length ratio (HW/TL). (D) Systolic blood pressure (SBP). (E) Diastolic blood pressure (DBP). (F) Blood glucose. Data represent mean ± SEM (*n* = 6). *p < .05, **p < .01, NS, not significant by Student unpaired *t*‐test (B, C) or 2‐way repeated‐measures ANOVA and Bonferroni post‐hoc tests (A, D through F).

We subjected the mice to chronic‐restraint stress for two weeks using a restrainer model (Figures S1A and 1B). The body weight (BW) and subcutaneous and inguinal adipose tissue weights were significantly lower in the S‐DPP4^+/+^ mice compared to the NS‐DPP4^+/+^ mice (Figures 1A,B and S1C). The ratio of heart weight to tibia length (HW/TL), systolic blood pressure (SBP), and blood glucose were increased in the S‐DPP4^+/+^ mice compared with the NS‐DPP4^+/+^ mice (Figure 1C,D,F). There was no significant difference in diastolic blood pressure (DBP) between the two groups (Figure 1E). As shown in Figure S2A, there was no significant difference in glucose tolerance between the NS‐DPP4^+/+^ and S‐DPP4^+/+^ mice. However, insulin tolerance was significantly worse in the S‐DPP4^+/+^ mice at all time points after 30 min (Figure S2B).

### 3.2 Effects of chronic stress on plasma DPP4 levels, pathological changes in cardiac tissue and cardiac function

**FIGURE 2:**
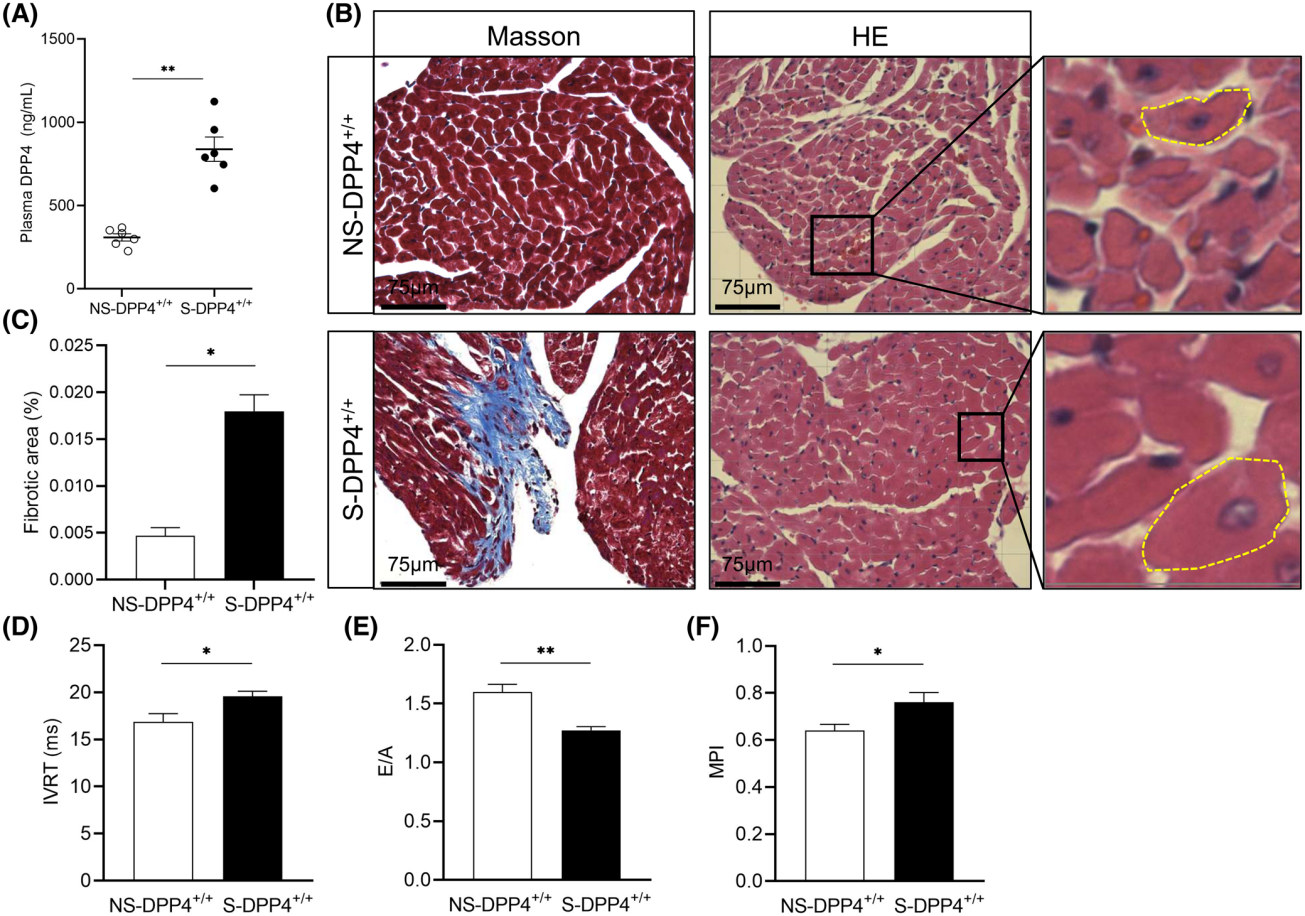
Changes in plasma DPP4 activity, pathological changes in cardiac tissue, and changes in cardiac function after chronic stress. (A) Plasma DPP4 levels. (B) HE and Masson's trichrome staining. Scale bar = 75 μm. (C) Quantification of the fibrotic area by Masson staining. (D–F) Echocardiographic parameters IVRT, E/A, and MPI in both experiment groups. Data represent mean ± SEM (*n* = 4–6). *p < .05, **p < .01 versus the NS‐DPP4^+/+^ group by Student unpaired *t*‐test (A, C through F).

The plasma DPP4 levels were significantly increased in the S‐DPP4^+/+^ group compared with the NS‐DPP4^+/+^ group (Figure 2A). HE staining showed hypertrophy of cardiomyocytes in the S‐DPP4^+/+^ group compared with the NS‐DPP4^+/+^ group (Figure 2B). Masson staining assays showed increased interstitial fibrosis in the heart tissue in the S‐DPP4^+/+^ group compared to the NS‐DPP4^+/+^ group (Figure 2B,C). Myocardial damage by stress was confirmed by HE and Masson's trichrome staining. Compared with the NS‐DPP4^+/+^ mice, IVRT and MPI were increased and E/A was decreased in the S‐DPP4^+/+^ (Figure 2D–F).

### 3.3 Effects of chronic stress on oxidative stress, inflammation, and proteolysis

**FIGURE 3:**
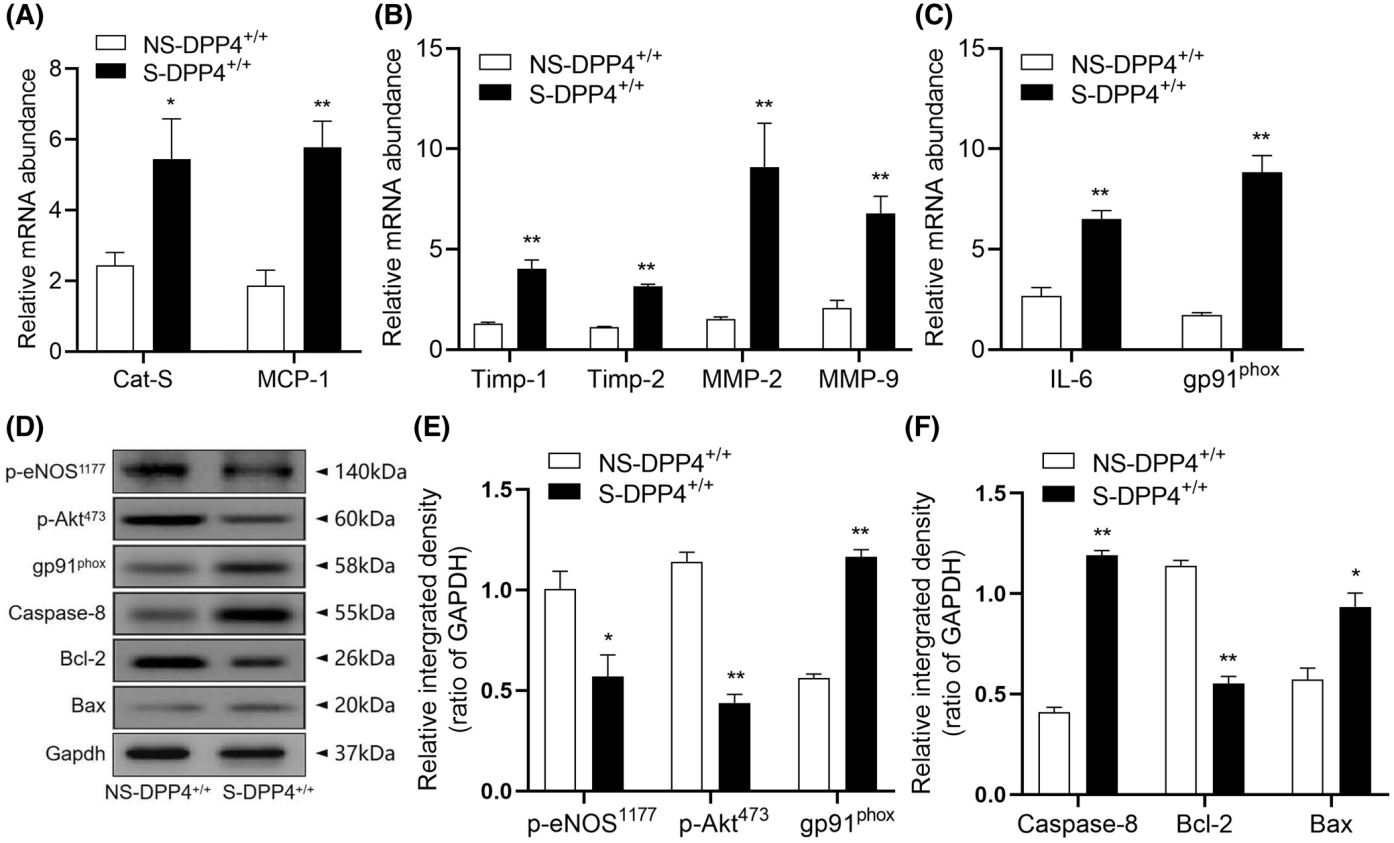
Two weeks of immobilization stress increased the levels of genes related to inflammation, oxidative stress, apoptosis, and proteolysis in cardiac tissues of stressed mice. (A–C) Gene expressions of CatS, MCP‐1, TIMP‐1, TIMP‐2, MMP‐2, MMP‐9, IL‐6, and gp91^phox^ based on quantitative real‐time PCR. An immunoblot analysis using equal amounts of total proteins from all samples was performed. (D–F) Representative Western blot images and quantitative data showing the levels of p‐eNOS^S1177^, p‐Akt^473^, gp91^phox^, caspase‐8, Bax, and Bcl‐2 proteins in the cardiac tissues of both groups. Results are mean ± SEM (*n* = 3–6). *p < .05, **p < .01 versus the NS‐DPP4^+/+^ group by Student's *t*‐test (A through C, E, F).

We analyzed the expression of genes related to oxidative stress and inflammation in cardiac tissues of NS‐DPP4^+/+^ and S‐DPP4^+/+^ mice by chronic stress after 14 days. The levels of oxidative stress (gp91^phox^) and inflammation‐related (IL‐6 and MCP‐1) genes were significantly elevated in mice from the S‐DPP4^+/+^ group compared with the NS‐DPP4^+/+^ group. We also observed an increase in the levels of proteolysis‐related (MMP‐2,‐9, TIMP‐1,‐2, and CatS) genes in myocardial tissues of mice in the S‐DPP4^+/+^ mice compared with the NS‐DPP4^+/+^ mice (Figure 3A–C). Immunoblot analysis was performed using equal amounts of proteins from all samples. Similarly, quantitative data from protein blotting showed elevated levels of gp91^phox^ and apoptosis‐related proteins (caspase‐8 and Bax) and decreased levels of p‐eNOS^S1177^, p‐Akt^473^, and anti‐apoptotic Bcl‐2 proteins in the myocardial tissues of mice in the S‐DPP4^+/+^ mice (Figure 3D–F).

### 3.4 Effects of DPP4 deficiency on body weight, adipose tissue weight, HW/TL, blood pressure, and blood glucose in stressed mice

**FIGURE 4:**
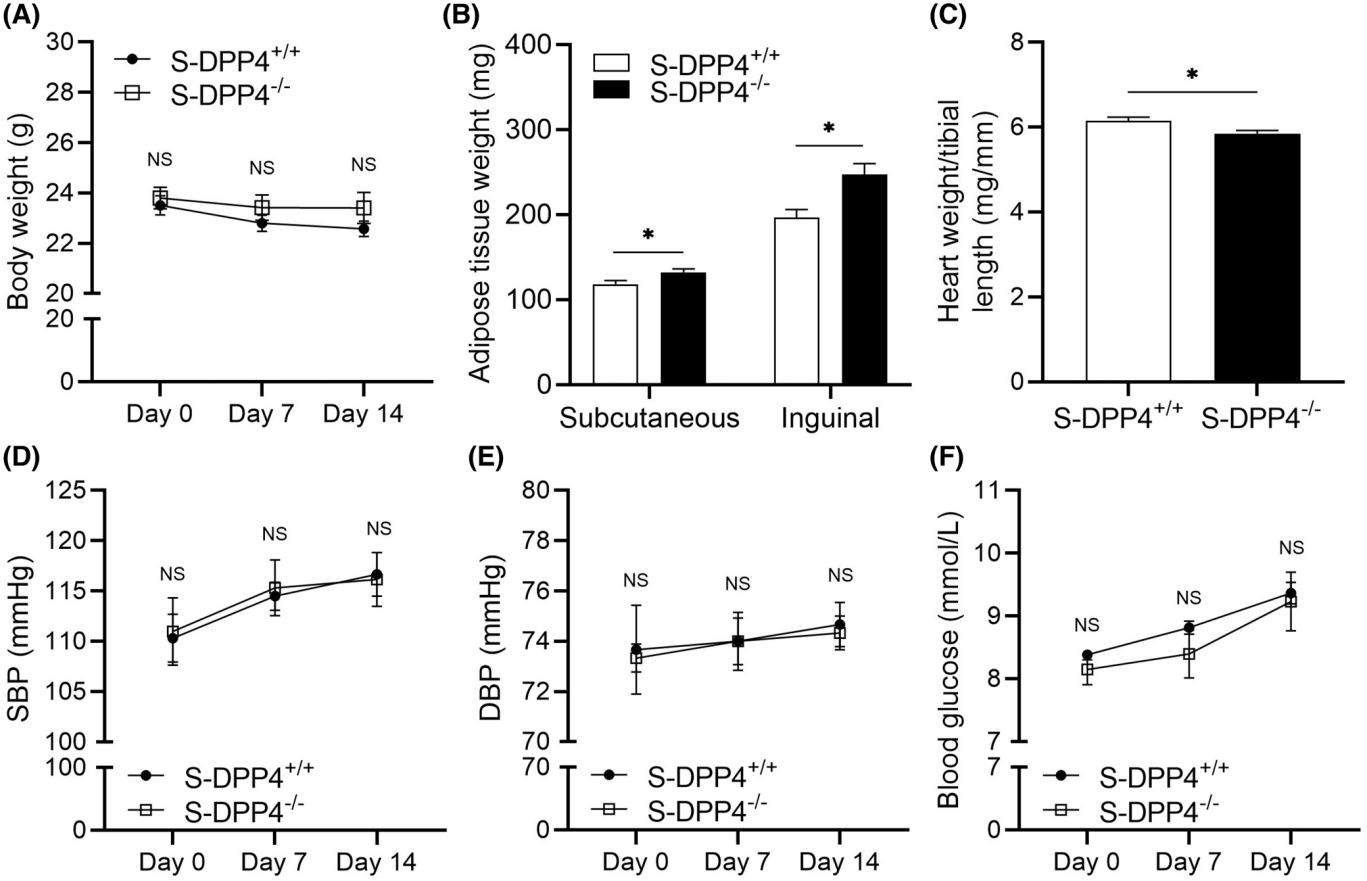
DPP4 deficiency increased adipose tissue weight and decreased HW/TL in stressed mice. (A) Effect of DPP4 deficiency on BW. (B) Effect of DPP4 deficiency on adipose tissue. (C) Quantification of the HW/TL ratio. (D) SBP changes in the stress and S‐DPP4^−/−^ groups. E DBP changes in the stress and S‐DPP4^−/−^ groups. (F) Blood glucose level. Data represent mean ± SEM (*n* = 6). *p < .05, NS, not significant by Student unpaired *t*‐test (B, C) or two‐way repeated‐measures ANOVA and Bonferroni post‐hoc tests (A, D through F).

To investigate the effect of DPP4 on stressed mice, we compared S‐DPP4^+/+^ and S‐DPP4^−/−^ mice. Compared with the S‐DPP4^+/+^ mice, subcutaneous and inguinal adipose tissue weights were significantly higher and HW/TL was significantly lower in the S‐DPP4^−/−^ mice (Figure 4B,C). DPP4^−/−^ markedly reduced the ratio of HW/TL in stressed mice, suggesting that DPP4^−/−^ improved left ventricular hypertrophy. The differences in BW, SBP, DBP, and blood glucose between the two groups were not statistically significant (Figure 4A,D–F). We also observed that there no significant difference in BW, SBP, DBP, HW/TL, and blood glucose between the NS‐DPP4^+/+^ and NS‐DPP4^−/−^ groups were not statistically significant at the baseline (Figure S3).

### 3.5 Effects of DPP4 deficiency on cardiac histopathological changes and cardiac function in stressed mice

**FIGURE 5:**
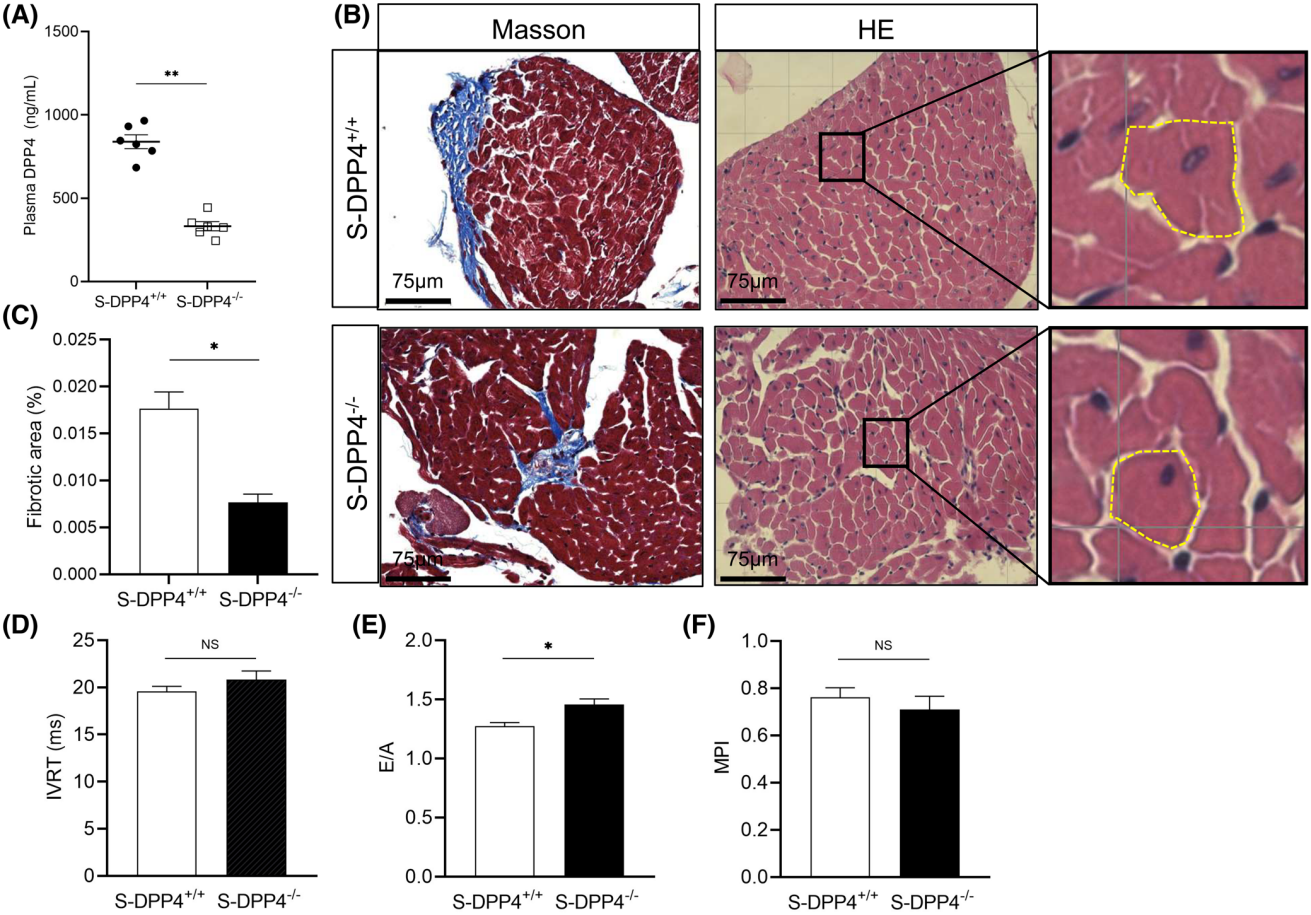
DPP4 deficiency resulted in reduced plasma DPP4 activity, cardiac hypertrophy, and fibrosis in chronically stressed mice. (A) Effect of chronic stress for two weeks on plasma DPP4 in DPP4‐deficient mice. (B) Representative images of HE and Masson's trichrome staining of cardiac tissue in the stress and S‐DPP4^−/−^ groups. The cardiomyocytes are stained red, and the collagenous fibers are stained blue. Scale bar = 75 μm. (C) Quantification of the fibrotic area by Masson staining in both experimental groups. (D–F) Levels of echocardiographic parameters IVRT, E/A, and MPI in experimental groups. Data represent mean ± SEM (*n* = 4–6). *p < .05, **p < .01, NS, no significant versus the S‐DPP4^+/+^ group by Student unpaired *t*‐test (A, C through F).

Plasma DPP4 activity was significantly reduced in the S‐DPP4^−/−^ group of mice compared with the S‐DPP4^+/+^ group (Figure 5A). HE staining showed that the structure of cardiomyocytes in the S‐DPP4^−/−^ group was more complete than that in the S‐DPP4^+/+^ group, and the arrangement of cardiomyocytes was more orderly, with clearer cell gaps (Figure 5B). Masson staining showed that myocardial fibrosis and collagen deposition were reduced in the S‐DPP4^−/−^ group compared with the S‐DPP4^+/+^ group (Figure 5B,C). E/A was significantly elevated in the S‐DPP4^−/−^ group compared to the S‐DPP4^+/+^ group, whereas the differences in IVRT and MPI were not significant (Figure 5D–F). At the baseline, with the exception of the plasma DPP4 levels, there was also no significant difference in fibrosis area, IVRT, E/A, and MPI between NS‐S‐DPP4^+/+^ and NS‐DPP4^−/−^ mice (Figure S4). Thus, the above findings confirm that DPP4^−/−^ may attenuate stress‐induced myocardial injury and diastolic dysfunction.

### 3.6 Effects of DPP4 deficiency on glucose metabolism, oxidative stress, inflammation, and protein hydrolysis in chronically stressed mice

**FIGURE 6:**
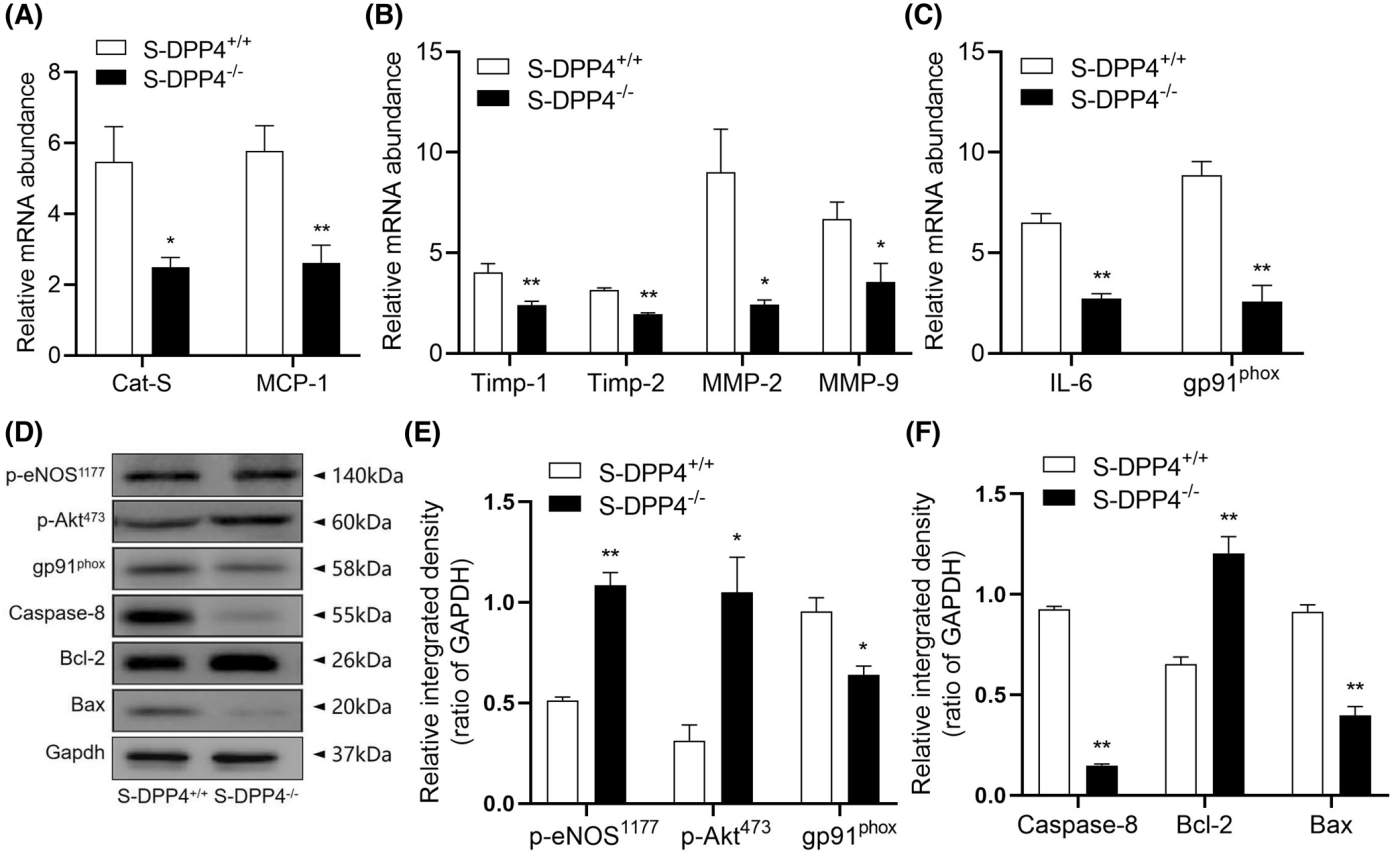
DPP4 deficiency attenuates inflammation, oxidative stress, apoptosis, and proteolysis‐related mRNA and protein levels in the cardiac tissues of stressed mice. (A–C) Gene expressions of CatS, MCP‐1, TIMP‐1, TIMP‐2, MMP‐2, MMP‐9, IL‐6, and gp91^phox^ by quantitative real‐time PCR. (D–F) Representative Western blot images and quantitative data showing the levels of p‐eNOS^S1177^, p‐Akt^473^, gp91^phox^, caspase‐8, Bax, and Bcl‐2 proteins in the cardiac tissues of both groups. Results are mean ± SEM (*n* = 3–6). *p < .05, **p < .01 versus the S‐DPP4^+/+^ group by Student's *t*‐test (A through C, E, F).

As anticipated, DPP4^−/−^ markedly improved insulin tolerance in mice under stress conditions, whereas it exhibited no effect on glucose tolerance in mice with and without stress (Figure S2C,D). Our observation showed that the levels of plasma corticosterone were higher in the S‐DPP4^+/+^ mice than those of the NS‐DPP4^+/+^ mice (124.0 ± 14.6 vs. 88.1 ± 10.2 ng/mL; *p* < .01); and this change was reversed in S‐DPP4^−/−^ mice (97.9 ± 9.3 vs. 124.0 ± 14.6, *p* < .05). Compared with the S‐DPP4^+/+^ group, mice in the S‐DPP4^−/−^ group had significantly lower levels of CatS, MCP‐1, TIMP‐1, TIMP‐2, MMP‐2, MMP‐9, IL‐6, and gp91^phox^ genes (Figure 6A–C). Deletion of DPP4 had similarly ameliorative effects on the deleterious changes induced by stress. Quantitative data from protein blotting showed markedly decreased levels of gp91^phox^ and apoptosis‐related proteins (caspase‐8 and Bax) and increased levels of p‐eNOS^S1177^, p‐Akt^473^, and anti‐apoptotic Bcl‐2 proteins in myocardial tissues of the S‐DPP4^−/−^ group (Figure 6D–F).

### 3.7 Effects of DPP4 inhibition on body weight, adipose tissue weight, HW/TL, blood pressure, and blood glucose in stressed mice

**FIGURE 7:**
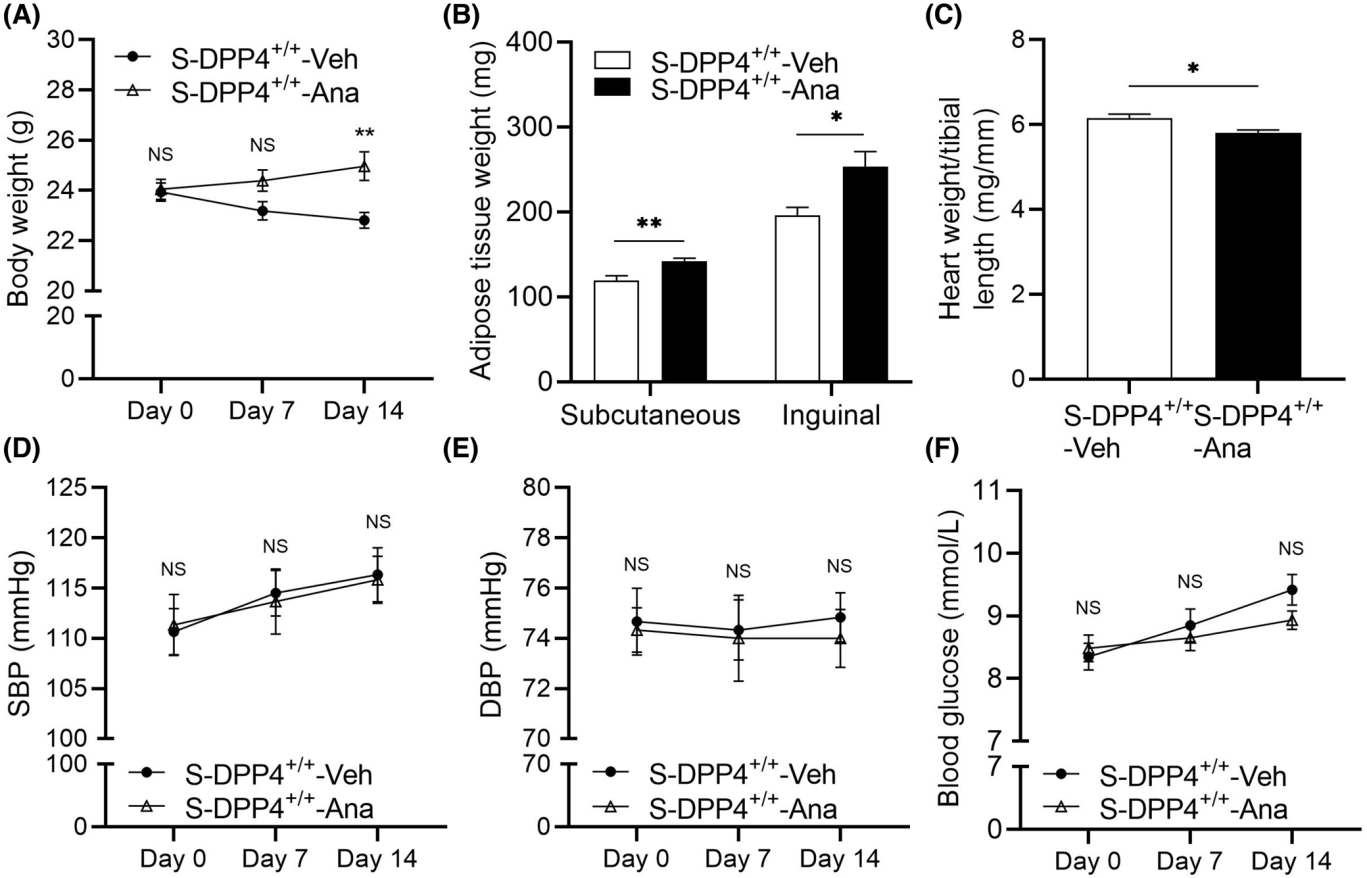
Inhibition of DPP4 increased BW and adipose tissue weight and decreased HW/TL in stressed mice. (A, B) Effect of DPP4 deficiency on BW and adipose tissue. (C) Quantification of the HW/TL ratio. (D, E) Changes in SBP and DBP in the stress and S‐Ana groups. (F) Blood glucose level. Data represent mean ± SEM (*n* = 6). *p < .05, **p < .01, NS, not significant by Student unpaired *t*‐test (B, C) or two‐way repeated‐measures ANOVA and Bonferroni post‐hoc tests (A, D through F).

To investigate the effect of DPP4 inhibition on stressed mice, we administered a gavage DPP4 inhibitor (anagliptin) for two weeks. The results revealed that mice in the S‐DPP4^+/+^‐Ana group had significantly increased BW and adipose tissue weight but significantly decreased HW/TL levels compared to the S‐DPP4^+/+^‐Veh group (Figure 7A–C). The differences in SBP, DBP, and blood glucose were not statistically significant (Figure 7D–F).

### 3.8 Effects of DPP4 inhibition on plasma DPP4 levels, cardiac histopathological changes, and cardiac function in stressed mice

**FIGURE 8:**
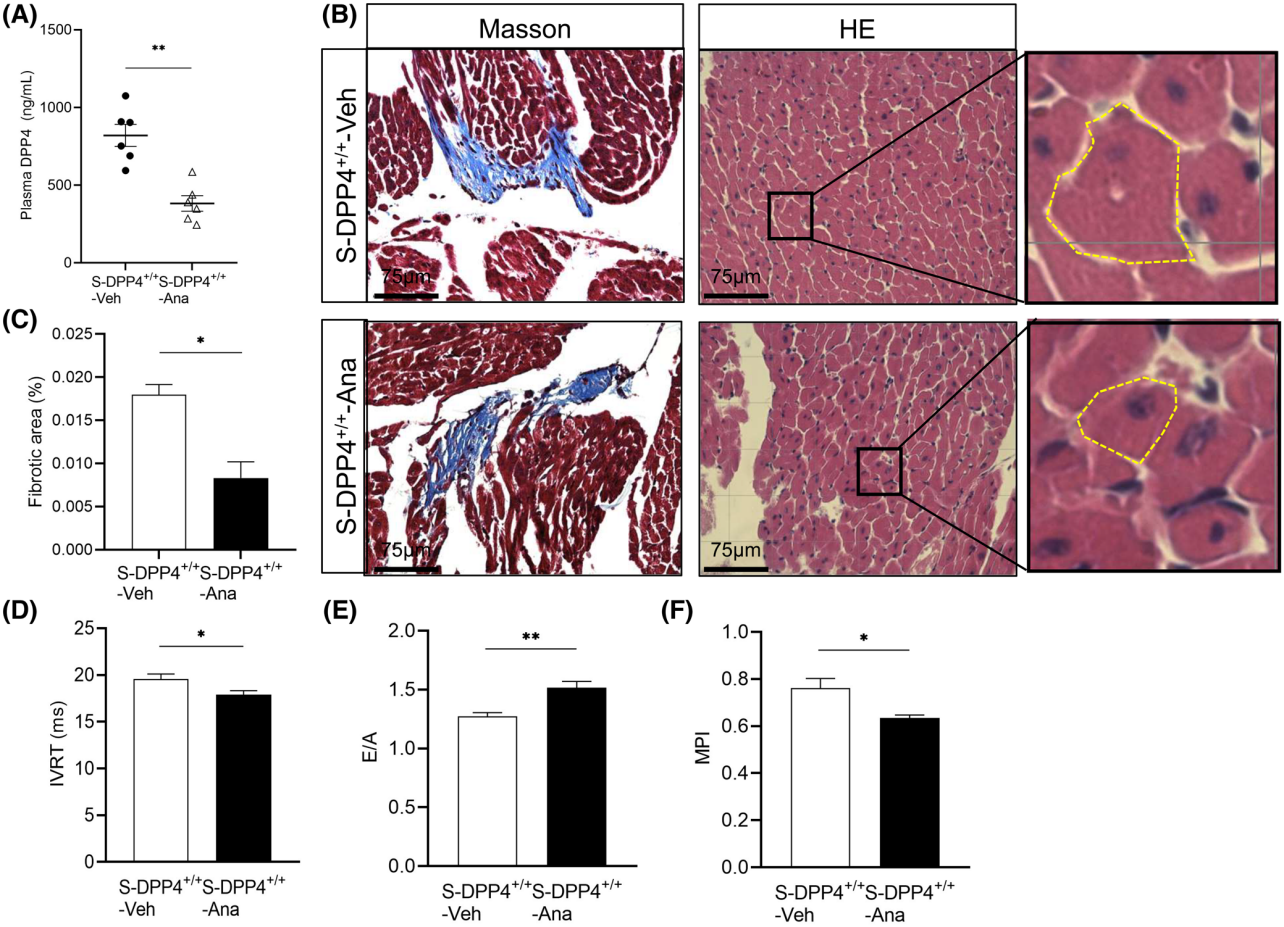
Inhibition of DPP4 attenuated plasma DPP4 activity, myocardial hypertrophy and fibrosis, and increased cardiac function in stressed mice. (A) Inhibition of DPP4 in stressed mice. (B) HE and Masson's trichrome staining. The cardiomyocytes were stained red, and the collagenous fibers were stained blue. Scale bar = 75 μm. (C) Quantification of fibrotic area by Masson staining. (D–F) Quantification of the echocardiographic parameters IVRT, E/A, and MPI in the experimental groups. Data represent mean ± SEM (*n* = 4–6). *p < .05, **p < .01 versus the S‐DPP4^+/+^‐Veh group by Student unpaired *t*‐test (A, C through F).

Plasma DPP4 activity was significantly reduced in the S‐DPP4^+/+^‐Ana mice compared with the S‐DPP4^+/+^‐Veh (Figure 8A). HE staining showed that the cardiomyocytes in the S‐DPP4^+/+^‐Ana mice were more complete in morphology, clearer in outline, and better aligned compared with those in the S‐DPP4^+/+^‐Veh (Figure 8B). Masson staining showed that myocardial fibrosis was less severe in the S‐DPP4^+/+^‐Ana group than in the S‐DPP4^+/+^‐Veh group, and collagen deposition was reduced (Figure 8B,C). Compared with the S‐DPP4^+/+^‐Veh mice, mice in the S‐DPP4^+/+^‐Ana mice had significantly lower IVRT and higher E/A, while the differences in MPI were not statistically significant (Figure 8D–F).

### 3.9 Effects of DPP4 inhibition on glucose metabolism, oxidative stress, inflammation, and protein hydrolysis in chronically stressed mice

**FIGURE 9:**
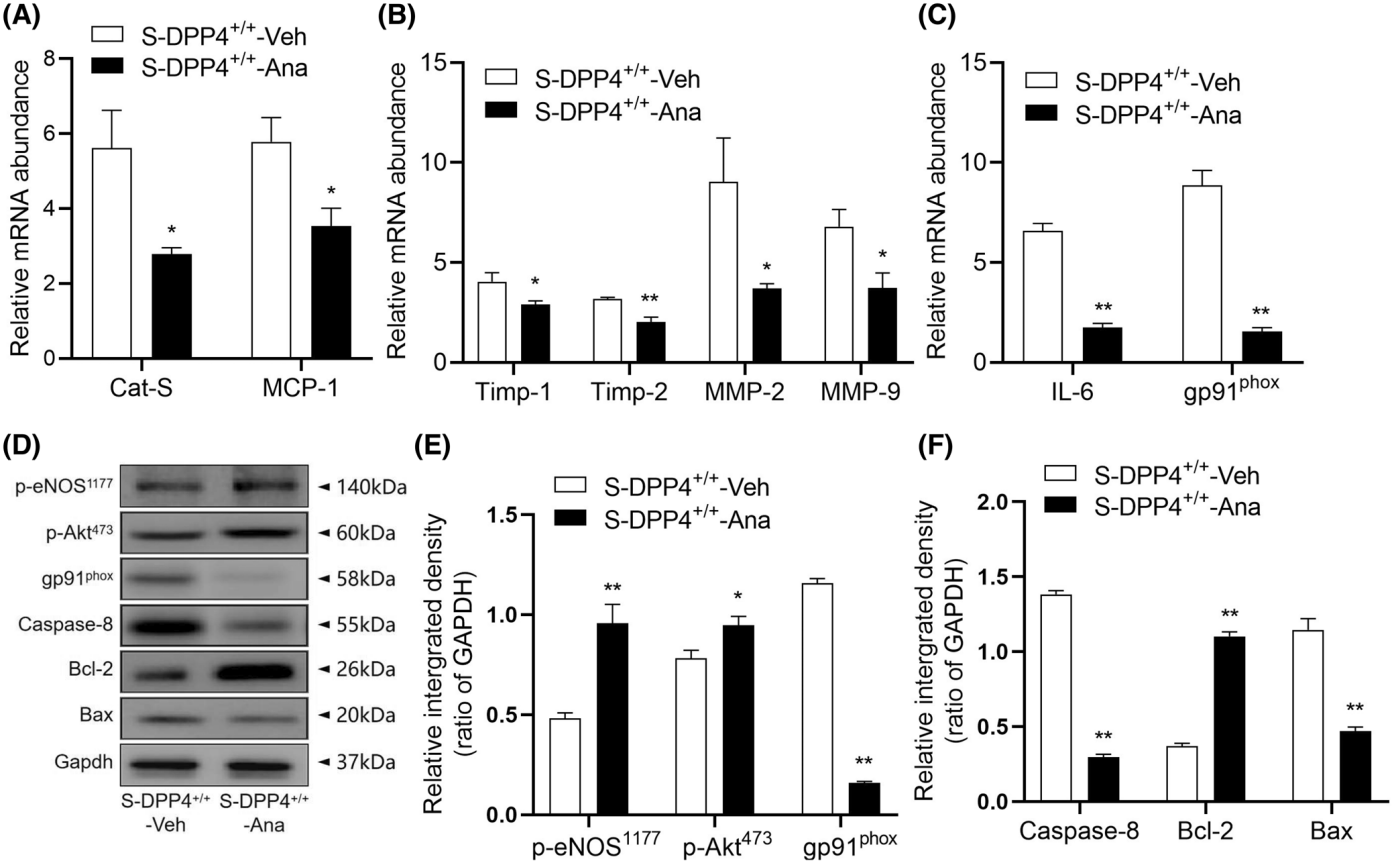
DPP4 inhibition attenuates inflammation, oxidative stress, apoptosis, and protein hydrolysis‐related mRNAs and protein levels in the cardiac tissues of stressed mice. (A–C) Gene expressions of CatS, MCP‐1, TIMP‐1, TIMP‐2, MMP‐2, MMP‐9, IL‐6, and gp91^phox^ by quantitative real‐time PCR. (D–F) Representative Western blot images and quantitative data showing the levels of p‐eNOS^S1177^, p‐Akt^473^, gp91^phox^, caspase‐8, Bax, and Bcl‐2 proteins in the cardiac tissues of both groups. Results are mean ± SEM (*n* = 3–6). *p < .05, **p < .01 versus the S‐DPP4^+/+^‐Veh group by Student's *t*‐test (A through C, E, F).

Similar to DPP4 deletion, pharmacological inhibition of DPP4 yielded the same effect on insulin tolerance and glucose tolerance (Figure S2E,F). Compared with the S‐DPP4^+/+^‐Veh group, the levels of CatS, MCP‐1, TIMP‐1, TIMP‐2, MMP‐2, MMP‐9, IL‐6, and gp91^phox^ genes were significantly reduced in the mice administered a DPP4 inhibitor (Figure 9A–C). Similarly, DPP4 inhibition had an ameliorative effect on the deleterious changes induced by stress. Quantitative protein blotting data showed decreased levels of gp91^phox^ and apoptosis‐related proteins (caspase‐8 and Bax) and increased levels of p‐eNOS^S1177^, p‐Akt^473^, and anti‐apoptotic Bcl‐2 proteins in myocardial tissues of mice in the S‐DPP4^+/+^‐Ana group (Figure 9D–F).

### 3.10 A DPP4 inhibitor attenuated oxidative stress‐induced apoptosis in cardiomyocytes

**FIGURE 10:**
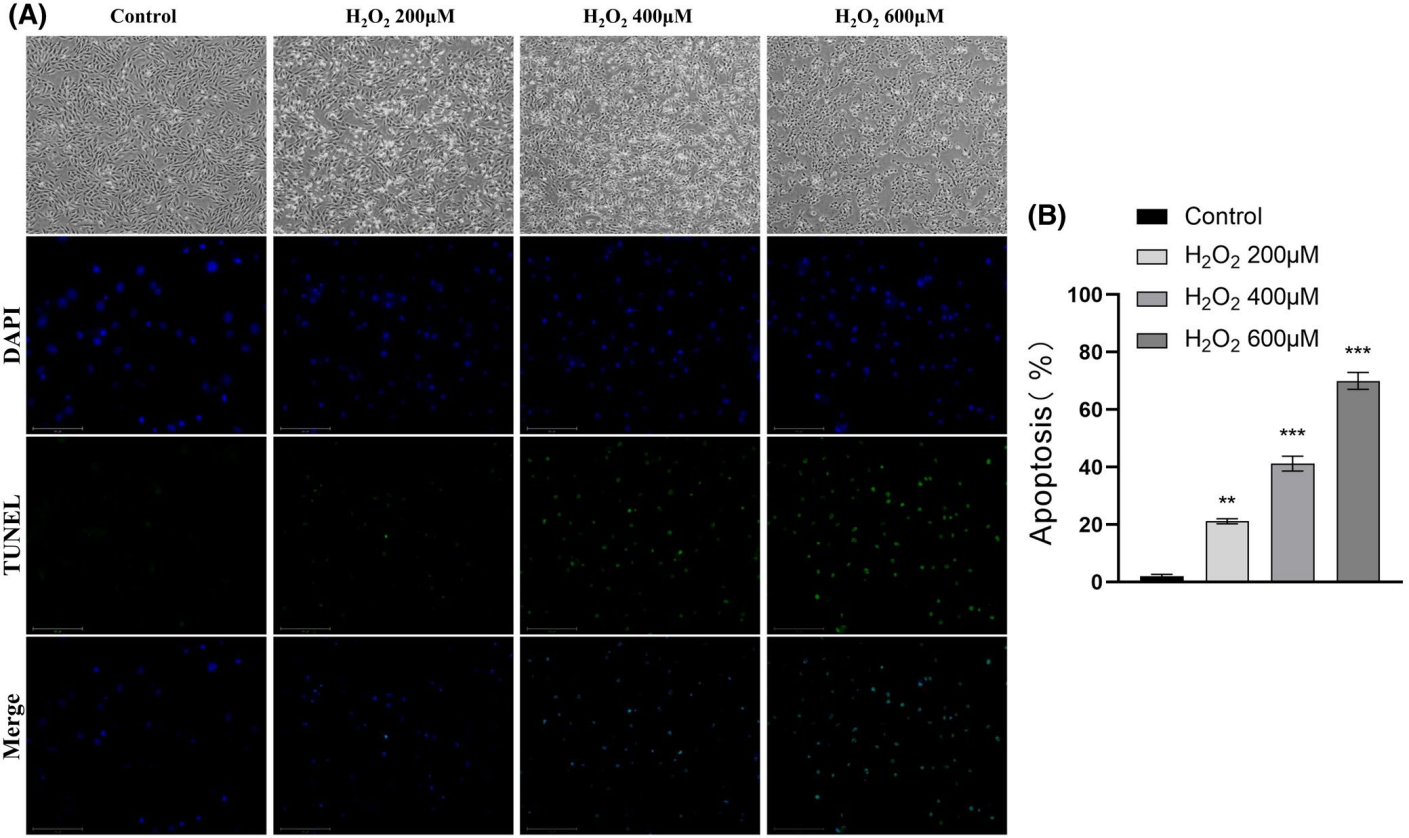
H_2_O_2_ increased oxidative stress and promoted apoptosis in H9c2 cardiomyocytes. (A) Effect of H_2_O_2_‐induced injury on the morphology of H9c2 cells (magnification, ×200) and TUNEL staining (bar = 100 μm). (B) The percentage of TUNEL‐positive cells. Results are mean ± SEM (*n* = 5–6). **p < .01, ***p < .001 versus the control by ANOVA and Tukey's post‐hoc tests (B).

**FIGURE 11:**
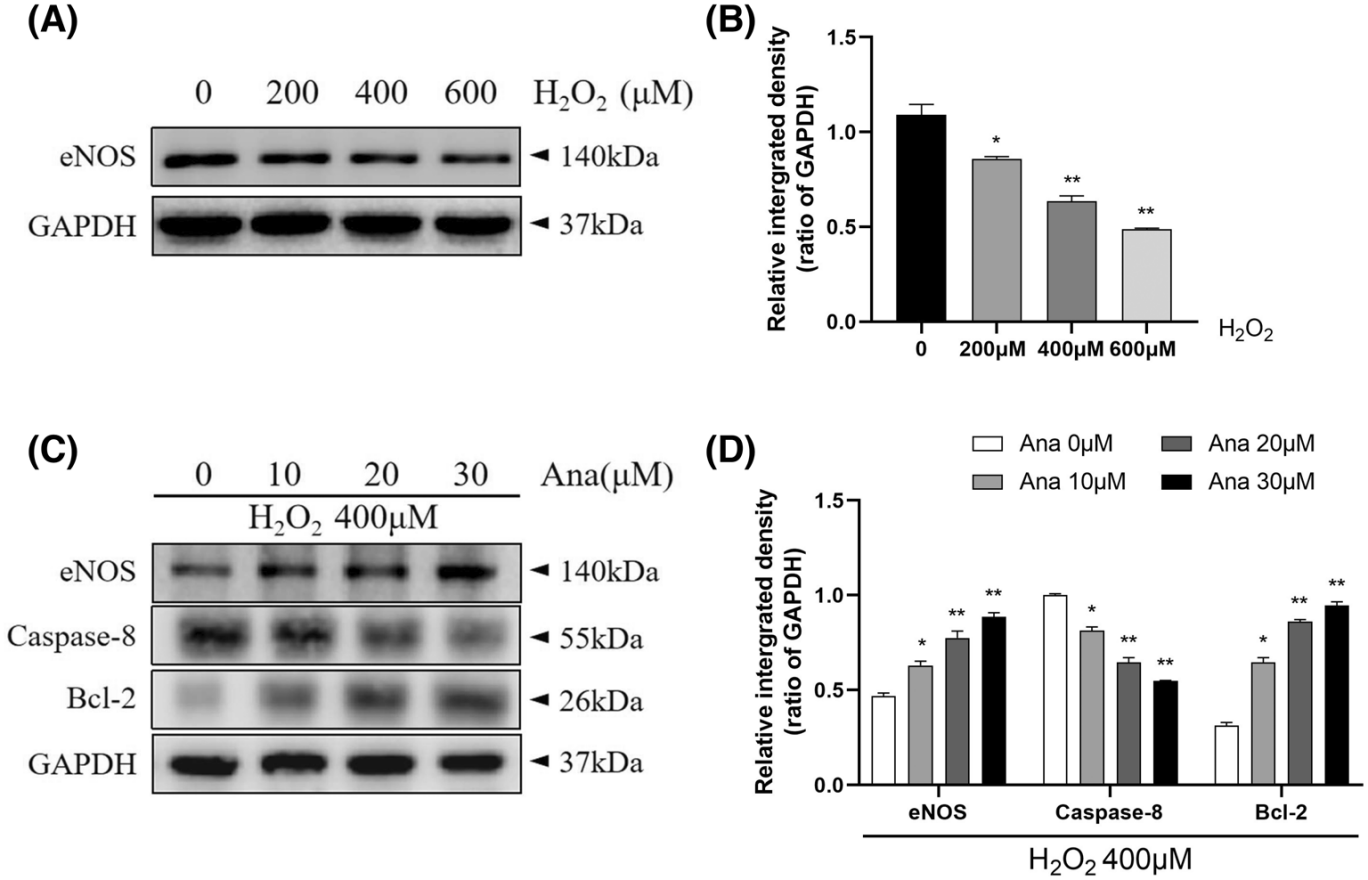
Effect of DPP4 inhibitor pretreatment on H_2_O_2_‐induced H9c2 cells. (A, B) H9c2 cell Western blot of the expression levels of eNOS under H_2_O_2_ treatment. (C, D) Western blot analysis of eNOS and apoptosis‐related (caspase‐8 and Bcl‐2) protein expression. Results are mean ± SEM (*n* = 3). *p < .05, **p < .01 versus the corresponding controls by ANOVA and Tukey's post‐hoc tests (B, D).

H9c2 cells were treated with graded hydrogen peroxide at 0, 200, 400, and 600 μM for 24 h. In the control group, the H9c2 cells were pike‐shaped or oval with defined cell outlines (Figure 10A; top panel). In contrast, cells in the H_2_O_2_ group were irregular and showed poor adhesion, shrinkage, a rounded shape, and partially incomplete cell membranes (Figure 10A; down panel). Cell damage and detachment were more obvious under the microscope. Approximately, 50% cell mortality was observed at the hydrogen peroxide concentration of 400 μM, so this concentration was used to establish a model of cardiomyocyte injury in the subsequent experiments. Treatment of H9c2 cells with H_2_O_2_ (400 μM for 24 h) significantly increased apoptosis and decreased eNOS protein expression (Figure 11A,B). Administration of a DPP4 inhibitor suppressed the hydrogen peroxide‐induced decrease in eNOS and Bcl‐2 protein expression, and decreased the expression of caspase‐8, which attenuated H_2_O_2_‐induced cellular damage (Figure 11C,D).

### 3.11 A DPP4 inhibitor activated the PI3K/AKT signaling pathway

**FIGURE 12:**
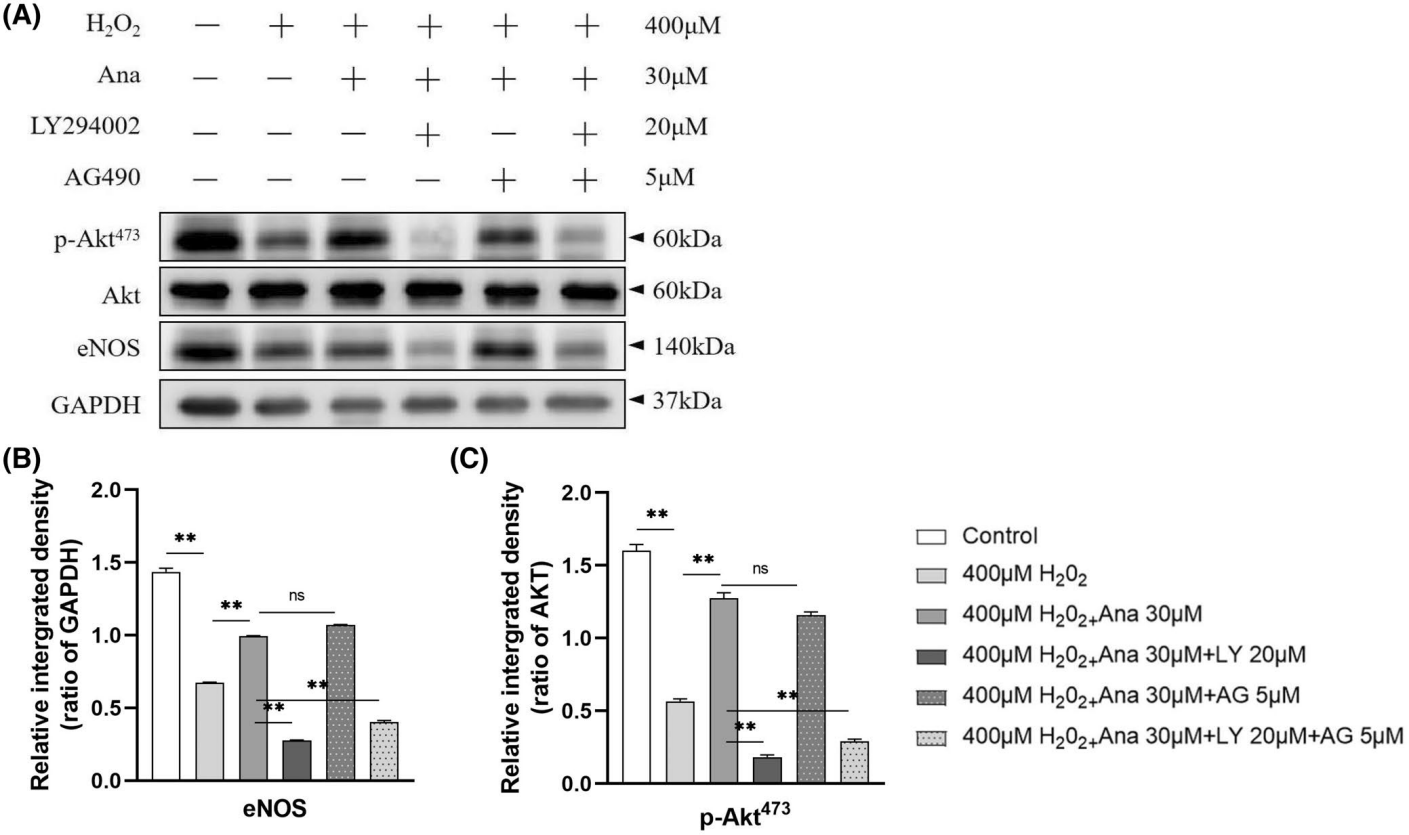
DPP4 inhibitor pretreatment activates PI3K/AKT signaling in H9c2 cells. (A–C) Effects of H_2_O_2_, anagliptin, LY294002, and AG490 on eNOS and p‐Akt^473^ proteins in H9c2 cells as analyzed by protein blotting. Data are presented as mean ± SEM (*n* = 3). *p < .05, **p < .01 versus the corresponding controls by ANOVA and Tukey's post‐hoc tests (B, C).

We determined the effects of H_2_O_2_, anagliptin, LY294002, and AG490 on eNOS and p‐Akt^473^ proteins by protein blotting. As shown in Figure 12A,B, H_2_O_2_ decreased the expressions of p‐Akt^473^ and eNOS, while the addition of anagliptin increased their expression, and further addition of LY294002 again decreased the expression of this protein. Addition of AG490 resulted in no significant changes. These findings suggest that anagliptin may activate the PI3K/AKT signaling pathway. The PI3K signaling pathway may be involved in mediating DPP‐4‐mediate beneficial effect (Figure 12).

## 4 DISCUSSION

We investigated the role of DPP4 in stress‐associated myocardial injury in mice, with special attention to oxidative stress and apoptosis in vivo and in vitro. In our study, we revealed for the first time the protective effects of DPP4 inhibition on the stress‐related myocardium and delved into its molecular mechanisms, including myocardial morphological and functional changes. We observed that DPP4 inhibition significantly attenuated oxidative stress, inflammation, apoptosis and protein hydrolysis and ameliorated myocardial injury under chronic stress conditions. The critical role of DPP4 inhibitor anagliptin in suppressing oxidative stress‐induced PI3K/AKT activity and attenuating stress‐induced eNOS expression was further demonstrated by cellular experiments.

Studies have shown that chronic exposure to restraint stress causes increased DPP4 levels, weight loss, and cardiac hypertrophy.^35^, ^36^, ^37^ Our results showed that plasma DPP4 levels increased, BW and groin and subcutaneous fat weight decreased, and heart weight/tibia length increased in mice after stress. At the same time, the increase in SBP and increase in blood glucose levels suggested an effect of chronic stress on overall metabolism. Myocardial fibrosis is characterized by increased accumulation of fibroblasts and excessive deposition of extracellular matrix proteins, inducing myocardial stiffness and ultimately cardiac dysfunction.^38^ Our study demonstrated that increased cardiomyocyte hypertrophy and collagen deposition were seen at the tissue level after stress, suggesting that chronic stress has an adverse effect on myocardial remodeling. Elevated levels of DPP4 may be associated with the metabolic changes such as blood glucose and insulin tolerance, providing a potential biomarker for metabolic abnormalities. We assessed cardiac function by echocardiographic parameters, and the increase in IVCT, IVRT, and MPI, as well as the decrease in E/A, in the stress group further corroborated the deterioration of cardiac function. Taken together, our results indicated that overactivation of DPP4 may play a detrimental role in regulating cardiac structure, function, and glucose metabolism. Encouragingly, both the DPP4 inhibitor group and the knockout group showed improvement on multiple levels, including insulin tolerance, myocardial structure, and function. It should be noted that we have shown that genetic and pharmacological interventions targeted toward DPP4 prevented the subcutaneous and inguinal adipose lost in stressed mice. Recently, we have demonstrated that both approaches can inhibit adipocyte de‐differentiation via the regulation of the glucagon‐like peptide‐1/adiponectin‐cathepsin K axis in mice under chronic stress conditions,^23^ suggesting that DPP4 inhibition may have applications for treating chronic stress‐related weight loss.

Oxidative stress has been recognized as a crucial factor in chronic stress myocardial injury.^39^ A large body of data confirms that oxidative stress is involved in the pathogenesis of several CVDs, including vascular injury and atherosclerosis.^40^ In cardiomyocytes, as in most other cell types, the major endogenous component of the antioxidant defense mechanism consists of antioxidant enzymes, where the steady‐state level of antioxidant molecules is largely dependent on the level of NADPH.^41^ The NADPH oxidase family is a complex enzyme system consisting of a variety of components, including the membrane‐bound cytochrome b‐558 (formed by heterodimers of gp91^phox^ and p22^phox^) and the cell membrane regulatory subunits p47^phox^ and p67^phox^.^42^ Of these, gp91^phox^, as the major isoform of NOX expressed in cardiomyocytes, plays an integral role in the regulation of oxidative stress.^43^ In the present study, gp91^phox^ expression was significantly upregulated in the stress group and showed a significant downregulation trend after DPP4 inhibition. This finding was further validated in the knockout group, indicating that DPP4 inhibitors can effectively inhibit oxidative stress, and also emphasizing the key role of DPP4 in chronic stress myocardial injury.

Endothelial NOS (eNOS) is present at high levels in cardiomyocytes, and eNOS in cardiomyocytes is beneficial for maintaining cardiovascular system function. Studies have shown that DPP4 inhibitors stimulate eNOS phosphorylation and activation by triggering the PI3K‐Akt pathway through upregulation of GLP‐1 levels.^44^ In the present study, we found that the expressions of both p‐eNOS and p‐Akt were significantly decreased in the stress group, whereas inhibition of DPP4 increased their expression, suggesting that DPP4 inhibitors may have a positive effect on the regulation of p‐eNOS^S1177^ and p‐Akt^473^. Cellular experiments confirmed the negative effect of oxidative stress on eNOS expression, which was reversed by the use of a DPP4 inhibitor. In addition, the PI3K inhibitor LY294002 was found to inhibit the amelioration of stress effects by the DPP4 inhibitor, whereas the Jak/Stat3 inhibitor AG490 had no effect. These results suggest that DPP4 inhibitors may be involved in the regulation of eNOS expression through the PI3K/Akt signaling pathway and may play a protective role in cardiomyocytes.

Apoptosis is one of the forms of programmed cell death and one of the key pathogenic mechanisms of chronic stress‐induced myocardial injury.^45^ In the context of chronic stress, caspase‐8, Bax, and Bcl‐2 play crucial roles as core genes in the regulation of apoptosis.^46^, ^47^, ^48^, ^49^ It has been shown that an imbalance of Bax and Bcl‐2 activates caspase‐8 and further leads to apoptosis.^50^ We found that caspase‐8 and Bax protein expression levels increased significantly after stress, while the Bcl‐2 expression levels decreased. Interestingly, the changes in the expression of the above genes showed diametrically opposite trends by inhibiting DPP4, with either a DPP4 inhibitor or DPP4 knockdown. This finding was further confirmed in cellular experiments: the expression of caspase‐8 decreased with increasing concentration gradient of the DPP4 inhibitor, while the expression of Bcl‐2 increased with increasing concentration gradient of the DPP4 inhibitor, suggesting that the activation of the apoptotic pathway by chronic stress could be inhibited by a DPP4 inhibitor.

Chronic stress increases the synthesis and release of inflammatory factors (IL‐6, MCP‐1) in tissues and blood, which in turn induces an inflammatory response.^51^, ^52^ In RNA analysis, we observed that the expressions of both IL‐6 and MCP‐1 tended to increase under chronic stress, whereas inhibition of DPP4 significantly attenuated the expression of inflammatory factors in cardiomyocytes, in contrast to the stress group. Cardiac matrix determines endothelial‐myocyte coupling and contractility in cardiomyocytes. Matrix metalloproteinases and their tissue inhibitors of metalloproteinases are important enzymes for metabolism of extracellular matrix proteins and regulate matrix degradation, thereby causing cardiac fibrosis and impairing myocardial function.^53^, ^54^ CatS is a cysteine protease localized in lysosomes in a variety of cardiovascular cells, such as cardiac fibroblasts, cardiomyocytes, vascular smooth muscle cells, and endothelial cells.^55^ CatS has been found to play an important role in the pathogenesis of myocardial remodeling and heart failure.^56^ We observed that chronic stress increased the gene expression of MMP‐2, MMP‐9, TIMP1, TIMP2, and CatS, suggesting their active role in myocardial remodeling. However, inhibition of DPP4 was able to slow down the process of myocardial remodeling.

A major potential limitation of the present study is that, although we provided data on genetic and pharmacological interventions targeting DPP4 to ameliorate chronic stress‐associated myocardial injury, we did not further analyze the aging‐ and sex‐dependent mechanism of action of DPP4 within the myocardium of mice under our experimental chronic stress conditions or the complex social stress conditions (e.g., a combination of social defeat and sensory interaction). In addition, we could not perform cardiomyocyte isolation to evaluate calcium‐handling abnormalities and in general to analyze sarcomere function from the genetic and pharmacological intervention mice/exposures. Further in vivo and in vitro studies will be needed to elucidate these issues.

Plasma DPP4 activity correlated with cardiac dysfunction in animal and human with heart failure.^57^ We observed an increase in DPP4 in the myocardium of mice under the chronic stress conditions and found that the use of a DPP4 inhibitor had a protective effect. This suggests that DPP4 inhibition may play an important role in stress CVD, as elevated DPP4 may serve as a molecular biomarker for this disease. This finding provides strong support for an in‐depth study of the pathogenesis of stress CVD and the development of related therapeutic strategies.

## AUTHOR CONTRIBUTIONS

**Zhe Jiang:** Conceptualization, Formal analysis, Investigation, Methodology, Writing – original draft. **Longguo Zhao:** Investigation, Data curation, Methodology. **Minglong Xin:** Investigation, Methodology. **Ying Wan:** Data curation, Methodology. **Shengnan Xu:** Formal analysis, Methodology, Resources. **Xueling Yue:** Validation. **Xianglan Jin:** Validation, Writing—review and editing. **Rihua Cui:** Validation, Writing—review and editing. **Yanglong Li:** Supervision, Writing—original draft. **Hongxian Wu:** Writing—original draft. **Weon Kim:** Validation, Writing—review and editing. **Xian Wu Cheng:** Conceptualization, Funding acquisition, Project administration, Supervision, Writing—original draft, Writing—review and editing. All authors read and approved the article.

## FUNDING INFORMATION

This work was supported in part by grants from the National Natural Science Foundation of China (nos. 81770485 and 82370424 to XWC; no. 82160103 to XJ).

## DISCLOSURES

The authors declare no potential conflicts of interest with respect to the research, authorship, and/or publication of this manuscript.

## ETHICS STATEMENT

The animal study protocols were approved by the Institutional Animal Care and Use Committees of Yanbian University (Protocol No. YD20211120005) and performed in accordance with the Guide for the Care and Use of Laboratory Animals published by the U.S. National Institutes of Health.

## Supporting information


Data S1.

## Data Availability

The data underlying this article will be shared upon reasonable request to the corresponding author.

## References

[1] Kivimäki M , Steptoe A . Effects of stress on the development and progression of cardiovascular disease. Nat Rev Cardiol. 2018;15:215‐229. 29213140 10.1038/nrcardio.2017.189

[2] Kivimäki M , Kawachi I . Work stress as a risk factor for cardiovascular disease. Curr Cardiol Rep. 2015;17:630. 26238744 10.1007/s11886-015-0630-8 PMC4523692

[3] Antoni MH , Dhabhar FS . The impact of psychosocial stress and stress management on immune responses in patients with cancer. Cancer. 2019;125:1417‐1431. 30768779 10.1002/cncr.31943 PMC6467795

[4] Reiche EM , Nunes SO , Morimoto HK . Stress, depression, the immune system, and cancer. Lancet Oncol. 2004;5:617‐625. 15465465 10.1016/S1470-2045(04)01597-9

[5] Osborne MT , Shin LM , Mehta NN , Pitman RK , Fayad ZA , Tawakol A . Disentangling the links between psychosocial stress and cardiovascular disease. Circ Cardiovasc Imaging. 2020;13:e010931. 32791843 10.1161/CIRCIMAGING.120.010931 PMC7430065

[6] Santosa A , Rosengren A , Ramasundarahettige C , et al. Psychosocial risk factors and cardiovascular disease and death in a population‐based cohort from 21 low‐, middle‐, and high‐income countries. JAMA Netw Open. 2021;4:e2138920. 34910150 10.1001/jamanetworkopen.2021.38920 PMC8674745

[7] Vaccarino V , Almuwaqqat Z , Kim JH , et al. Association of mental stress‐induced myocardial ischemia with cardiovascular events in patients with coronary heart disease. JAMA. 2021;326:1818‐1828. 34751708 10.1001/jama.2021.17649 PMC8579237

[8] Mifsud KR , Reul J . Mineralocorticoid and glucocorticoid receptor‐mediated control of genomic responses to stress in the brain. Stress. 2018;21:389‐402. 29614900 10.1080/10253890.2018.1456526

[9] Noushad S , Ahmed S , Ansari B , Mustafa UH , Saleem Y , Hazrat H . Physiological biomarkers of chronic stress: a systematic review. Int J Health Sci. 2021;15:46‐59. PMC8434839 34548863

[10] Piao L , Li Y , Narisawa M , Shen X , Cheng XW . Role of dipeptidyl peptidase‐4 in atherosclerotic cardiovascular disease in humans and animals with chronic stress. Int Heart J. 2021;62:470‐478. 33994495 10.1536/ihj.20-181

[11] Sher LD , Geddie H , Olivier L , et al. Chronic stress and endothelial dysfunction: mechanisms, experimental challenges, and the way ahead. Am J Physiol Heart Circ Physiol. 2020;319:H488‐H506. 32618516 10.1152/ajpheart.00244.2020

[12] Thornberry NA , Gallwitz B . Mechanism of action of inhibitors of dipeptidyl‐peptidase‐4 (DPP‐4). Best Pract Res Clin Endocrinol Metab. 2009;23:479‐486. 19748065 10.1016/j.beem.2009.03.004

[13] Valencia I , Vallejo S , Dongil P , et al. DPP4 promotes human endothelial cell senescence and dysfunction via the PAR2‐COX‐2‐TP axis and NLRP3 inflammasome activation. Hypertension. 2022;79:1361‐1373. 35477273 10.1161/HYPERTENSIONAHA.121.18477

[14] Shigeta T , Aoyama M , Bando YK , et al. Dipeptidyl peptidase‐4 modulates left ventricular dysfunction in chronic heart failure via angiogenesis‐dependent and ‐independent actions. Circulation. 2012;126:1838‐1851. 23035207 10.1161/CIRCULATIONAHA.112.096479

[15] Lee SY , Wu ST , Du CX , Ku HC . Potential role of dipeptidyl peptidase‐4 in regulating mitochondria and oxidative stress in cardiomyocytes. Cardiovasc Toxicol. 2024;24:1090‐1104. 38955919 10.1007/s12012-024-09884-z

[16] Jin X , Jin C , Nakamura K , et al. Increased dipeptidyl peptidase‐4 accelerates chronic stress‐related thrombosis in a mouse carotid artery model. J Hypertens. 2020;38:1504‐1513. 32205561 10.1097/HJH.0000000000002418

[17] Baggio LL , Drucker DJ . Biology of incretins: GLP‐1 and GIP. Gastroenterology. 2007;132:2131‐2157. 17498508 10.1053/j.gastro.2007.03.054

[18] Soejima H , Ogawa H , Morimoto T , et al. Dipeptidyl peptidase‐4 inhibitors reduce the incidence of first cardiovascular events in Japanese diabetic patients. Heart Vessel. 2023;38:1371‐1379. 10.1007/s00380-023-02291-4 37522902

[19] Bethel MA , Sourij H , Stevens SR , et al. Time‐dependent event accumulation in a cardiovascular outcome trial of patients with type 2 diabetes and established atherosclerotic cardiovascular disease. Cardiovasc Diabetol. 2023;22:72. 36978066 10.1186/s12933-023-01802-x PMC10054031

[20] Dicembrini I , Monami M , Mannucci E . Dypeptidylpeptidase‐4 inhibitors and the cardiovascular system: how to manage the fil rouge. Nutr Metab Cardiovasc Dis. 2019;29:215‐219. 30718142 10.1016/j.numecd.2018.12.009

[21] White WB , Cannon CP , Heller SR , et al. Alogliptin after acute coronary syndrome in patients with type 2 diabetes. N Engl J Med. 2013;369:1327‐1335. 23992602 10.1056/NEJMoa1305889

[22] Scirica BM , Bhatt DL , Braunwald E , et al. Saxagliptin and cardiovascular outcomes in patients with type 2 diabetes mellitus. N Engl J Med. 2013;369:1317‐1326. 23992601 10.1056/NEJMoa1307684

[23] Zhang M , Yue X , Xu S , et al. Dipeptidyl peptidase‐4 disturbs adipocyte differentiation via the negative regulation of the glucagon‐like peptide‐1/adiponectin‐cathepsin K axis in mice under chronic stress conditions. FASEB J. 2024;38:e23684. 38795334 10.1096/fj.202400158R

[24] Zhu E , Hu L , Wu H , et al. Dipeptidyl peptidase‐4 regulates hematopoietic stem cell activation in response to chronic stress. J Am Heart Assoc. 2017;6:e006394. 28710180 10.1161/JAHA.117.006394 PMC5586325

[25] Yang G , Lei Y , Inoue A , et al. Exenatide mitigated diet‐induced vascular aging and atherosclerotic plaque growth in ApoE‐deficient mice under chronic stress. Atherosclerosis. 2017;264:1‐10. 28734203 10.1016/j.atherosclerosis.2017.07.014

[26] Xin M , Jin X , Cui X , et al. Dipeptidyl peptidase‐4 inhibition prevents vascular aging in mice under chronic stress: modulation of oxidative stress and inflammation. Chem Biol Interact. 2019;314:108842. 31586451 10.1016/j.cbi.2019.108842

[27] Lei Y , Yang G , Hu L , et al. Increased dipeptidyl peptidase‐4 accelerates diet‐related vascular aging and atherosclerosis in ApoE‐deficient mice under chronic stress. Int J Cardiol. 2017;243:413‐420. 28549747 10.1016/j.ijcard.2017.05.062

[28] Piao L , Zhao G , Zhu E , et al. Chronic psychological stress accelerates vascular senescence and impairs ischemia‐induced neovascularization: the role of dipeptidyl peptidase‐4/glucagon‐like Peptide‐1‐adiponectin axis. J Am Heart Assoc. 2017;6:e006421. 28963101 10.1161/JAHA.117.006421 PMC5721852

[29] Xu S , Piao L , Wan Y , et al. CTSS modulates stress‐related carotid artery thrombosis in a mouse FeCl(3) model. Arterioscler Thromb Vasc Biol. 2023;43:e238‐e253. 37128920 10.1161/ATVBAHA.122.318455

[30] Meng X , Huang Z , Inoue A , et al. Cathepsin K activity controls cachexia‐induced muscle atrophy via the modulation of IRS1 ubiquitination. J Cachexia Sarcopenia Muscle. 2022;13:1197‐1209. 35098692 10.1002/jcsm.12919 PMC8978007

[31] Lin Z , Zhao M , Zhang X , et al. CD8^+^ T‐cell deficiency protects mice from abdominal aortic aneurysm formation in response to calcium chloride2. J Hypertens. 2024;42:1966‐1975. 39146540 10.1097/HJH.0000000000003823 PMC11451972

[32] Yue X , Piao L , Wang H , et al. Cathepsin K deficiency prevented kidney damage and dysfunction in response to 5/6 nephrectomy injury in mice with or without chronic stress. Hypertension. 2022;79:1713‐1723. 35726642 10.1161/HYPERTENSIONAHA.122.19137 PMC9278705

[33] Fang J , Shu S , Dong H , et al. Histone deacetylase 6 controls cardiac fibrosis and remodelling through the modulation of TGF‐β1/Smad2/3 signalling in post‐infarction mice. J Cell Mol Med. 2024;28:e70063. 39232846 10.1111/jcmm.70063 PMC11374528

[34] Wang H , Meng X , Piao L , et al. Cathepsin S deficiency mitigated chronic stress‐related neointimal hyperplasia in mice. J Am Heart Assoc. 2019;8:e011994. 31296090 10.1161/JAHA.119.011994 PMC6662117

[35] Martí O , Martí J , Armario A . Effects of chronic stress on food intake in rats: influence of stressor intensity and duration of daily exposure. Physiol Behav. 1994;55:747‐753. 8190805 10.1016/0031-9384(94)90055-8

[36] Mao M , Deng Y , Wang L , et al. Chronic unpredictable mild stress promotes atherosclerosis via adipose tissue dysfunction in ApoE^−/−^ mice. PeerJ. 2023;11:e16029. 37692113 10.7717/peerj.16029 PMC10484201

[37] McLellan MA , Skelly DA , Dona MSI , et al. High‐resolution transcriptomic profiling of the heart during chronic stress reveals cellular drivers of cardiac fibrosis and hypertrophy. Circulation. 2020;142:1448‐1463. 32795101 10.1161/CIRCULATIONAHA.119.045115 PMC7547893

[38] Fontes MT , Arruda‐Junior DF , Dos Santos DS , et al. Dipeptidyl peptidase 4 inhibition rescues PKA‐eNOS signaling and suppresses aortic hypercontractility in male rats with heart failure. Life Sci. 2023;323:121648. 37001807 10.1016/j.lfs.2023.121648

[39] Lv H , He Y , Wu J , Zhen L , Zheng Y . Chronic cold stress‐induced myocardial injury: effects on oxidative stress, inflammation and pyroptosis. J Vet Sci. 2023;24:e2. 36726274 10.4142/jvs.22185 PMC9899938

[40] Shao R , Chen R , Zheng Q , et al. Oxidative stress disrupts vascular microenvironmental homeostasis affecting the development of atherosclerosis. Cell Biol Int. 2024;48:1781‐1801. 39370593 10.1002/cbin.12239

[41] Wang X , Zhang G , Dasgupta S , et al. ATF4 protects the heart from failure by antagonizing oxidative stress. Circ Res. 2022;131:91‐105. 35574856 10.1161/CIRCRESAHA.122.321050 PMC9351829

[42] Elnakish MT , Hassanain HH , Janssen PM , Angelos MG , Khan M . Emerging role of oxidative stress in metabolic syndrome and cardiovascular diseases: important role of Rac/NADPH oxidase. J Pathol. 2013;231:290‐300. 24037780 10.1002/path.4255

[43] Vermot A , Petit‐Härtlein I , Smith SME , Fieschi F . NADPH oxidases (NOX): an overview from discovery, molecular mechanisms to physiology and pathology. Antioxidants Basel. 2021;10:890. 34205998 10.3390/antiox10060890 PMC8228183

[44] Moon JY , Woo JS , Seo JW , et al. The dose‐dependent organ‐specific effects of a dipeptidyl peptidase‐4 inhibitor on cardiovascular complications in a model of type 2 diabetes. PLoS One. 2016;11:e0150745. 26959365 10.1371/journal.pone.0150745 PMC4784786

[45] Li HR , Zheng XM , Liu Y , et al. L‐carnitine alleviates the myocardial infarction and left ventricular remodeling through Bax/Bcl‐2 signal pathway. Cardiovasc Ther. 2022;2022:9615674. 35692375 10.1155/2022/9615674 PMC9150988

[46] Meng LB , Shan MJ , Yu ZM , et al. Chronic stress: a crucial promoter of cell apoptosis in atherosclerosis. J Int Med Res. 2020;48:300060518814606. 30700193 10.1177/0300060518814606 PMC7140195

[47] Wan Y , Piao L , Xu S , et al. Cathepsin S deficiency improves muscle mass loss and dysfunction via the modulation of protein metabolism in mice under pathological stress conditions. FASEB J. 2023;37:e23086. 37428652 10.1096/fj.202300395RRR

[48] Wang H , Inoue A , Lei Y , Wu H , Hong L , Cheng XW . Cathepsins in the extracellular space: focusing on non‐lysosomal proteolytic functions with clinical implications. Cell Signal. 2023;103:110531. 36417977 10.1016/j.cellsig.2022.110531

[49] Wan Y , Piao L , Xu S , et al. Cathepsin S activity controls chronic stress‐induced muscle atrophy and dysfunction in mice. Cell Mol Life Sci. 2023;80:254. 37589754 10.1007/s00018-023-04888-4 PMC10435624

[50] Zhang H , Wei M , Sun N , Wang H , Fan H . Melatonin attenuates chronic stress‐induced hippocampal inflammatory response and apoptosis by inhibiting ADAM17/TNF‐α axis. Food Chem Toxicol. 2022;169:113441. 36162616 10.1016/j.fct.2022.113441

[51] Fleshner M . Stress‐evoked sterile inflammation, danger associated molecular patterns (DAMPs), microbial associated molecular patterns (MAMPs) and the inflammasome. Brain Behav Immun. 2013;27:1‐7. 22964544 10.1016/j.bbi.2012.08.012

[52] Scally C , Abbas H , Ahearn T , et al. Myocardial and systemic inflammation in acute stress‐induced (Takotsubo) cardiomyopathy. Circulation. 2019;139:1581‐1592. 30586731 10.1161/CIRCULATIONAHA.118.037975 PMC6438459

[53] Mishra PK , Givvimani S , Chavali V , Tyagi SC . Cardiac matrix: a clue for future therapy. Biochim Biophys Acta. 2013;1832:2271‐2276. 24055000 10.1016/j.bbadis.2013.09.004 PMC4111554

[54] Aggeli C , Pietri P , Felekos I , et al. Myocardial structure and matrix metalloproteinases. Curr Top Med Chem. 2012;12:1113‐1131. 22519443 10.2174/1568026611208011113

[55] Cheng XW , Shi GP , Kuzuya M , Sasaki T , Okumura K , Murohara T . Role for cysteine protease cathepsins in heart disease: focus on biology and mechanisms with clinical implication. Circulation. 2012;125:1551‐1562. 22451605 10.1161/CIRCULATIONAHA.111.066712

[56] Cheng XW , Narisawa M , Wang H , Piao L . Overview of multifunctional cysteinyl cathepsins in atherosclerosis‐based cardiovascular disease: from insights into molecular functions to clinical implications. Cell Biosci. 2023;13:91. 37202785 10.1186/s13578-023-01040-4 PMC10197855

[57] dos Santos L , Salles TA , Arruda‐Junior DF , et al. Circulating dipeptidyl peptidase IV activity correlates with cardiac dysfunction in human and experimental heart failure. Circ Heart Fail. 2013;6:1029‐1038. 23894014 10.1161/CIRCHEARTFAILURE.112.000057

