# Supplementary material for: Dipeptidyl peptidase‐4 deficiency prevents chronic stress‐induced cardiac remodeling and dysfunction in mice
Source: FASEB J. 2025 Feb 19;39(4):e70398. doi: 10.1096/fj.202402328R (PMC11836924; doi:10.1096/fj.202402328R)
Supplement: Supplementary file 1 — Data S1. [file FSB2-39-e70398-s001.pdf]

Figure S1

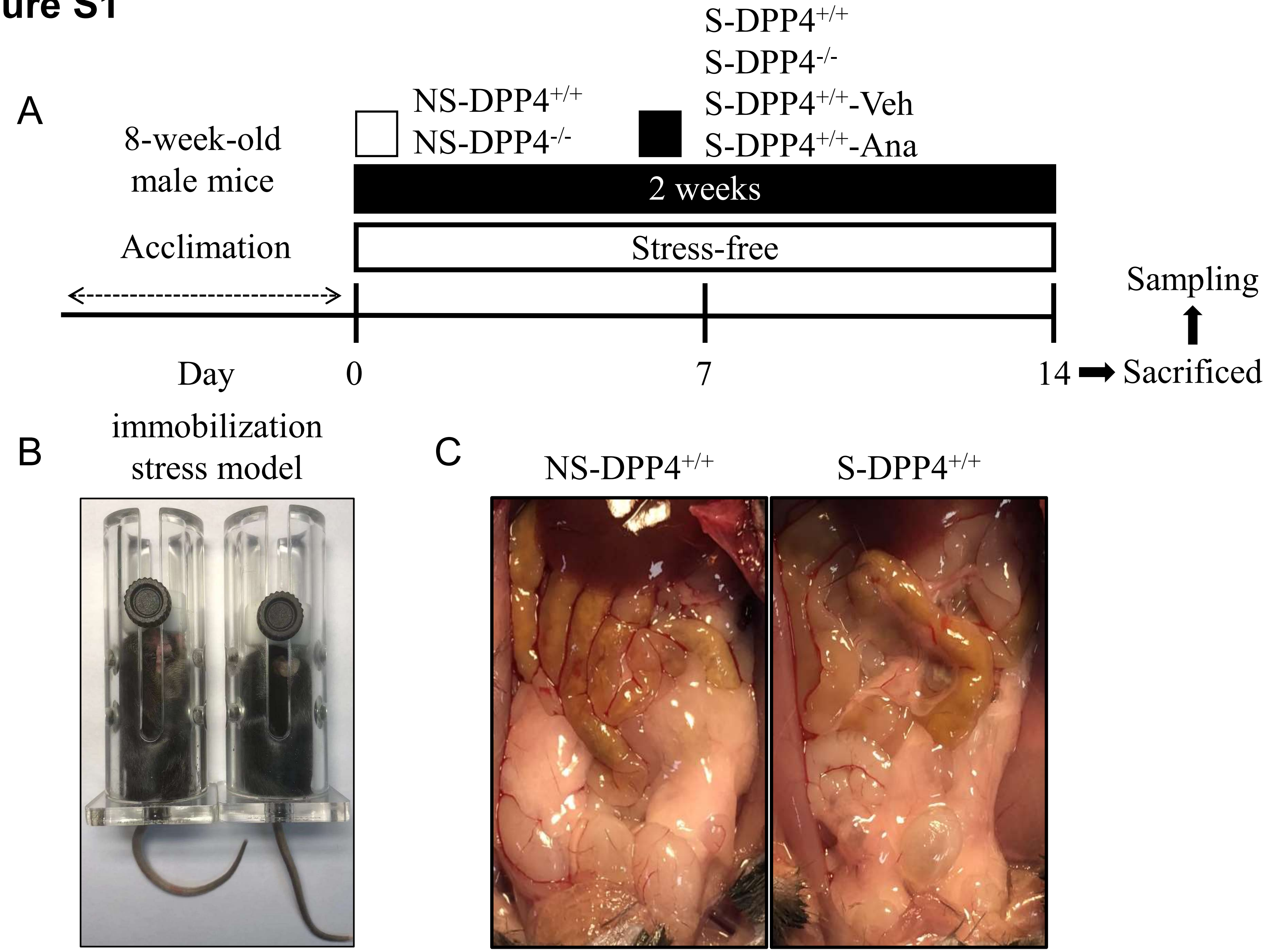

Figure S1：Experimental design and timeline in restraint-stressed mice. A. Timeline diagram depicting the restraint stress-induction protocol. B. The chronic immobilization stress animal model. C. Representative adipose tissue visualizations.

Figure S2

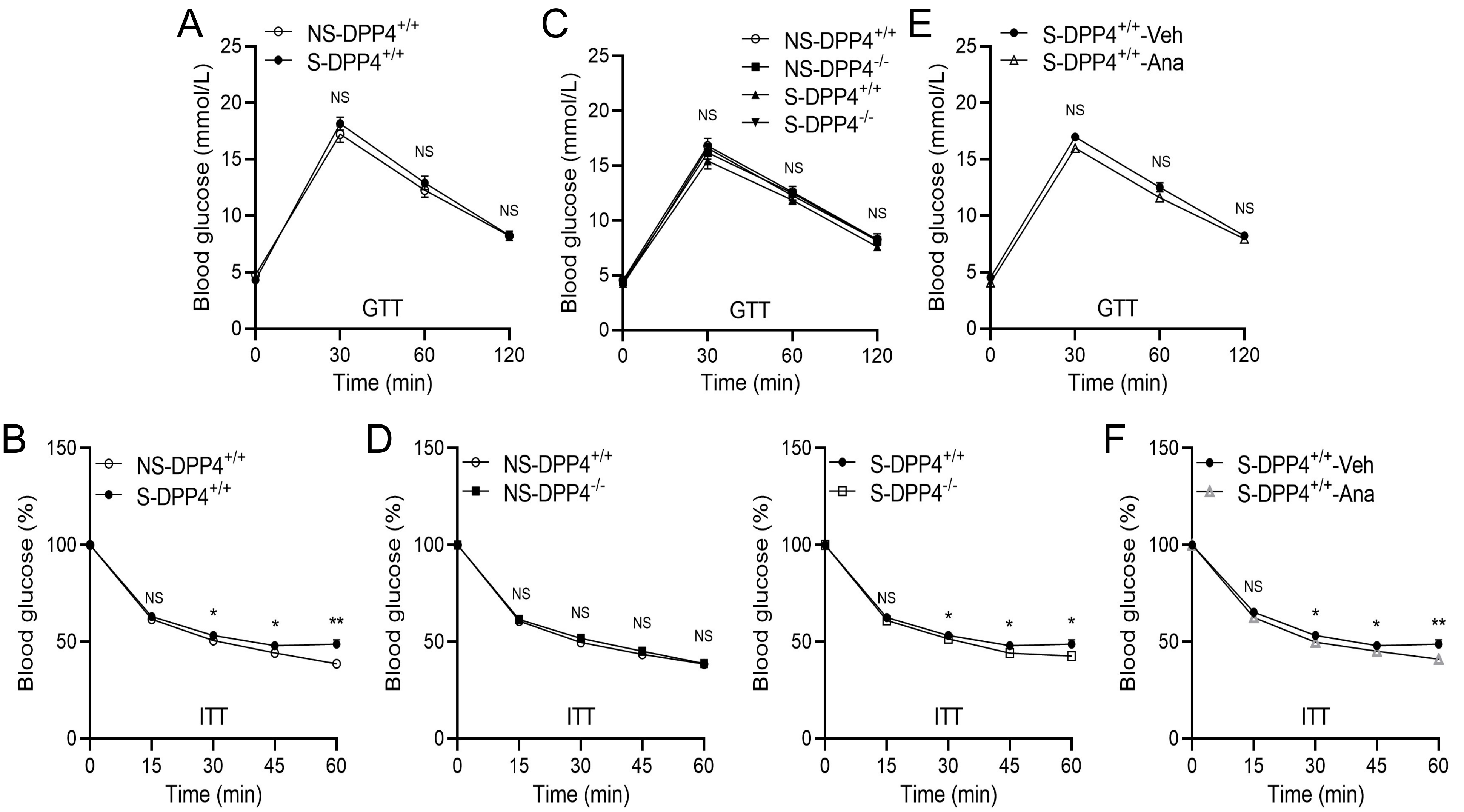

Figure S2：After 2 weeks of stress, the changes in GTT and ITT in the experimental groups. A、C、E：Comparison of GTT in two and four groups of mice. B、D、F：Comparison of ITT in two groups of mice. Data represent mean ± SEM (n=6). *p<0.05, **p<0.01 by 2-way repeated-measures ANOVA and Bonferroni post hoc tests (A through F).

Figure S3

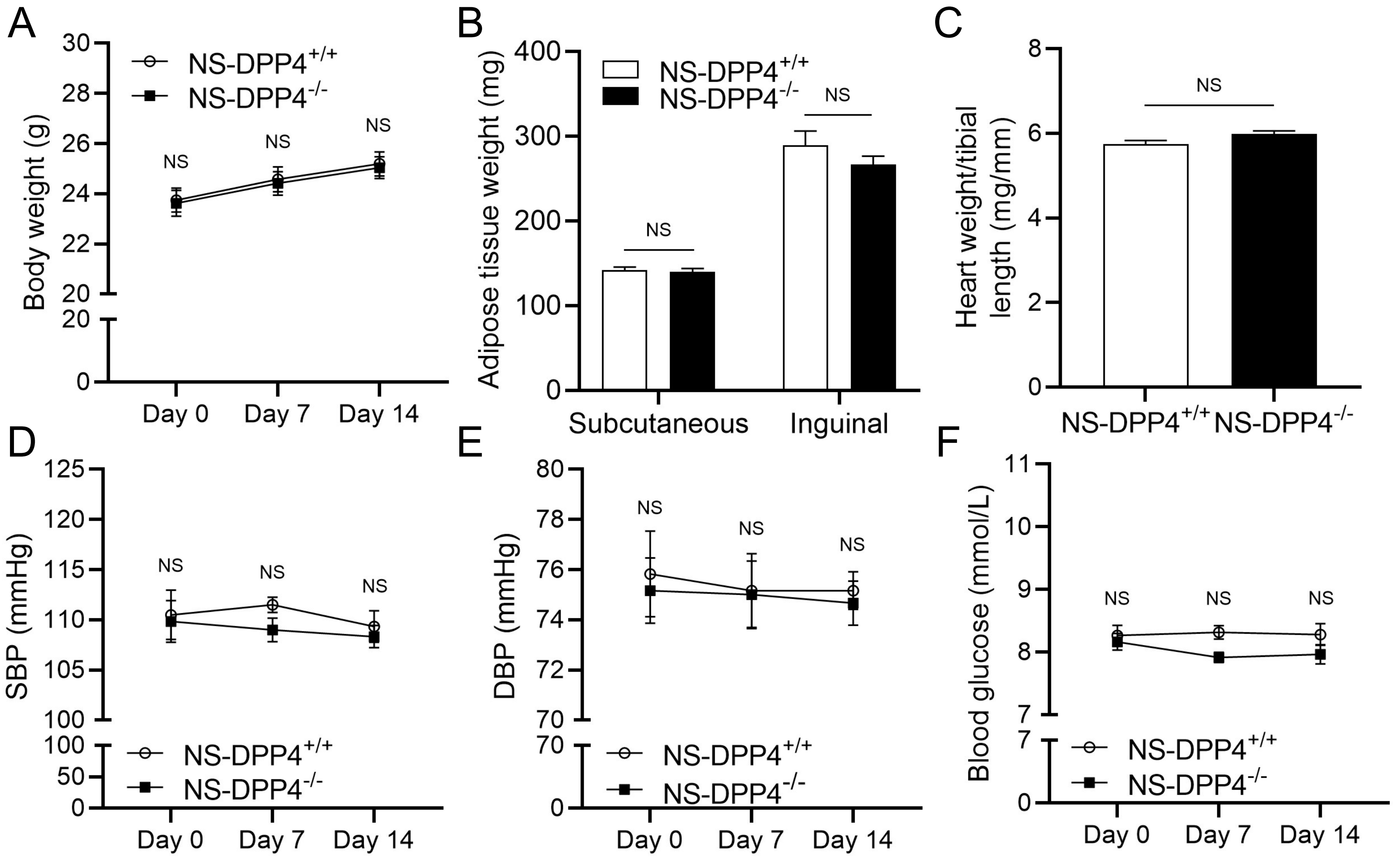

Figure S3： Variations in the parameters between the NS-DPP4$^{+/+}$ and NS-DPP4$^{-/-}$ groups. A. Changes in body weight. B. Weight of subcutaneous and inguinal adipose tissue. C. Heart weight to tibia length ratio. D. SBP. E. DBP. F. Blood glucose levels. Data represent mean ± SEM (n=4-6). *p<0.05, **p<0.01 by Student unpaired t test (B, C) or 2-way repeated-measures ANOVA and Bonferroni post hoc tests (A, D though F).

Figure S4

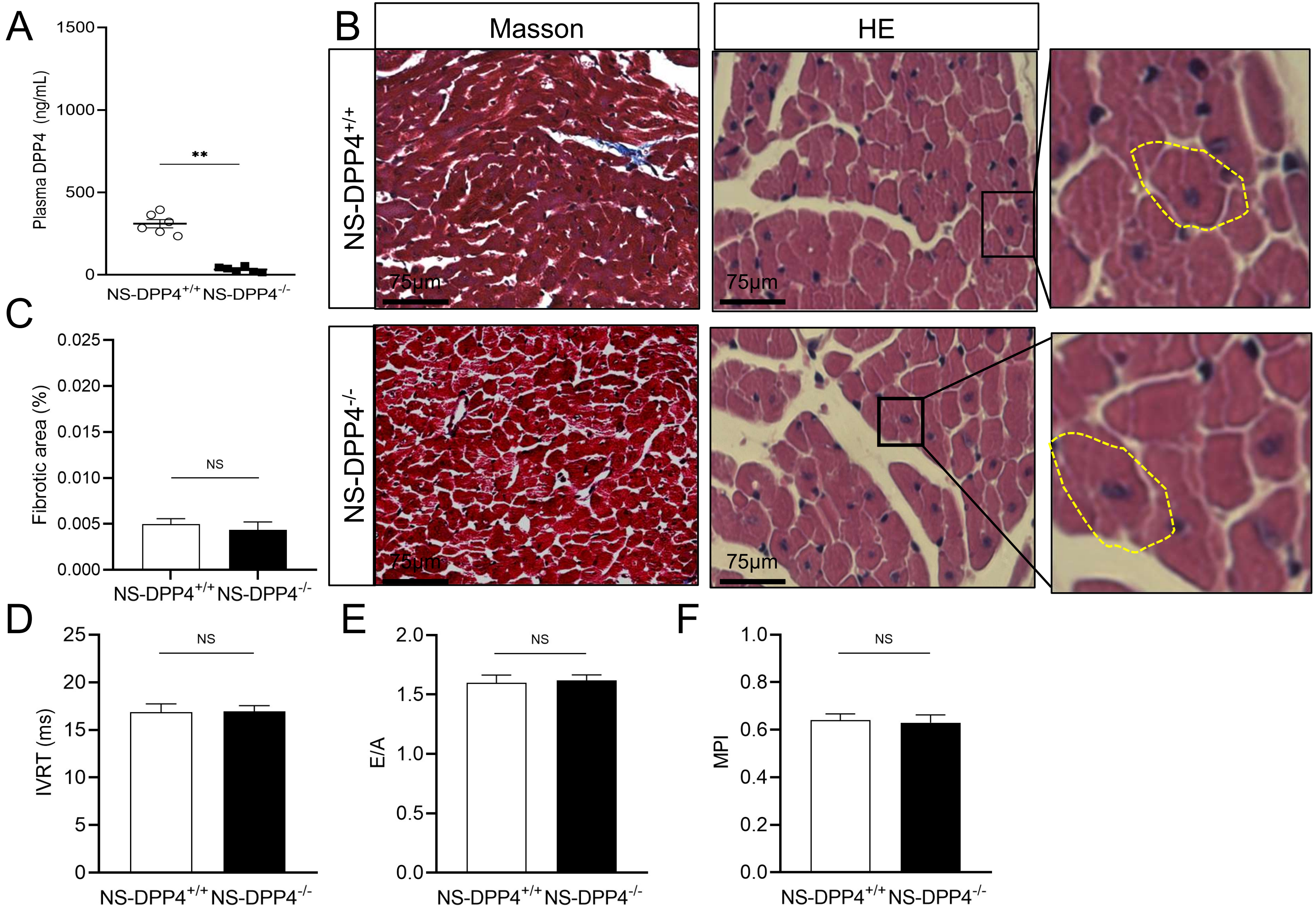

Figure S4: The changes in Plasma DPP4 levels, cardiac fibrosis, and cardiac function in the NS-DPP4$^{+/+}$ and NS-DPP4$^{-/-}$ mice. A. Plasma DPP4 levels. B and C. Representative images and quantitative data for cardiac interstitial fibrosis. Scale bar = 75 μm. D–F. Quantification IVRT, E/A ratio, and MPI in the experimental groups. Data represent mean ± SEM (n=4-6). *p<0.05, **p<0.01 by Student's *t*-test (A, C, D-F).

Table 1. primer sequences used for real-time fluorescence quantitative PCR

Gene	Forward Primer	Reverse Primer
gp91^{phox}	ACTTTCCATAAGATGGTAGCTTGG	GCATTCACACACCACTCAACG
MCP-1	GCCCCACTCACCTGCTGCTACT	CCTGCTCGTGGTGATCCTCTTGT
MMP-2	CCCCATGAAGCCTTGTTTACC	TTGTAGGAGGTGCCCTGGAA
MMP-9	CCAGACGCTCTTCGAGAACC	GTTATAGAAGTGGCGGTTGT
TIMP-1	GCCTACACCCCAGTCATGGA	GGCCCGTGATGAGAAACTCTT
TIMP-2	GTCCCATGATCCCTTGCTACA	TGCCCATTGATGCTCTTCTCT
CatS	GTGGCCACTAAAGGGCCTG	ACCGCTTTTGTAGAAGAAGAAGGAG
IL-6	CCAAGAGGTGAGTGCTTCCC	CTGTTGTTCAGACTCTCTCCCT
GAPDH	ATGTGTCCGTCGTGGATCTGA	ATGCCTGCTTCACCACCTTCT
